# *Sphingomonas paucimobilis*‐Driven Epithelial–Endothelial Transition in Adenomyosis Pathogenesis

**DOI:** 10.1002/advs.202516652

**Published:** 2026-03-20

**Authors:** Peigen Chen, Hao Shi, Junxian He, Ziyu Liu, Yuanyuan Zhu, Xiaoyan Liang, Guihua Liu, Xing Yang

**Affiliations:** ^1^ Reproductive Medicine Center The Sixth Affiliated Hospital Sun Yat‐sen University Guangzhou China; ^2^ GuangDong Engineering Technology Research Center of Fertility Preservation Guangzhou China; ^3^ Biomedical Innovation Center The Sixth Affiliated Hospital Sun Yat‐sen University Guangzhou China

**Keywords:** adenomyosis, epithelial–endothelial transition, sphingomonas paucimobilis

## Abstract

Adenomyosis is a common gynecological disorder with incompletely understood pathogenesis. Through single‐cell RNA sequencing of adenomyosis tissues, we identified epithelial–endothelial transition (EET) as a key pathological mechanism and discovered transitional cells co‐expressing epithelial (EPCAM) and endothelial (PECAM1) markers. Using CSI‐Microbes pipeline and 2bRAD‐M validation, we found significant enrichment of *Sphingomonas paucimobilis* in adenomyosis lesions with spatial co‐localization to EET cells. Mouse experiments confirmed that vaginal inoculation with live *S. paucimobilis* recapitulated adenomyosis development, indicating that viable bacteria are required. Systematic inhibitor experiments validated a TNFα → NF‐κB → MMP signaling cascade: TNFα neutralization and NF‐κB inhibition blocked pathway activation and EET, while MMP inhibition attenuated downstream phenotypic changes. Protein‐level validation by Western blot and immunofluorescence across human tissue, mouse models, and cell culture confirm EET marker changes. This study elucidates a novel microbial‐driven cellular transformation mechanism and identifies actionable therapeutic targets for adenomyosis.

## Graphical abstract

This study identifies epithelial–endothelial transition (EET) as a novel adenomyosis pathogenic mechanism driven by *Sphingomonas paucimobilis* colonization. Systematic inhibitor experiments validate a TNFα→NF‐κB→MMP signaling cascade essential for EET. Critically, bacterial culture supernatant fails to induce pathological changes, demonstrating viable bacteria are required. Protein‐level validation across human tissue, mouse models, and cell culture confirms this microbial‐driven transformation.

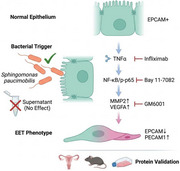

## 1 Introduction

Adenomyosis affects 20%–30% of women of reproductive age, making it one of the most common gynecological disorders [1, 2]. This condition involves the abnormal invasion of endometrial glands and stroma into the myometrium, often causing debilitating symptoms such as heavy menstrual bleeding, severe pelvic pain, and infertility [3]. These symptoms can profoundly impact a woman's quality of life and reproductive potential. While clinicians increasingly recognize adenomyosis as a significant health concern, we still lack a complete understanding of what drives this disease. The etiology appears highly variable between individuals, with genetic, hormonal, and immune factors all playing roles [4, 5]. Without clearer mechanistic insights, developing more effective treatments remains challenging.

In recent years, the role of the microbiome in female reproductive system diseases has gained increasing attention. Research has demonstrated that dysbiosis of the reproductive tract microbial communities is closely associated with the onset and progression of various gynecological conditions [6]. In patients with adenomyosis, cervical microbiota composition exhibits distinctive alterations, suggesting potential microbial involvement in disease pathological processes [7]. Furthermore, changes in vaginal microbiome diversity have been found to correlate with clinical manifestations of adenomyosis [8]. These emerging findings indicated that microbiome dysbiosis might represent a crucial component in adenomyosis pathogenesis, providing novel perspectives for understanding disease development and progression.

Beyond microbial dysbiosis, accumulating evidence suggests that cellular plasticity processes such as epithelial–mesenchymal transition (EMT) and related hybrid states contribute to uterine remodeling and gynecologic disease. In adenomyosis and endometriosis, multiple groups have reported upregulation of EMT‐related transcription factors and loss of epithelial adhesion molecules in eutopic and ectopic endometrium, together with enhanced migration, invasion and fibrosis [9, 10, 11, 12]. Recent work further highlights that dysregulated EMT programs in the endometrium are intertwined with extracellular matrix remodeling and inflammatory signaling, providing a mechanistic link between chronic injury, aberrant repair and lesion formation [10, 11, 12].

The hallmark of adenomyosis is endometrial invasion into the myometrium. Recent spatial transcriptomic analyses have uncovered distinct cellular and molecular features within these invasive lesions [13], while detailed examination of the endometrial‐myometrial junction reveals the complexity of this invasion process [14]. Together, prior studies on EMT in adenomyosis/endometriosis and on vasculogenic mimicry or endothelial‐like transitions in gynecologic cancers suggest that epithelial plasticity and acquisition of endothelial traits may be a recurrent theme in uterine pathology. However, whether a bona fide epithelial–endothelial transition (EET) occurs in adenomyosis, and how such a process might be regulated in vivo, has not been clarified.

Interestingly, microbes within tissues can regulate invasive behavior in other diseases. Anaerobic bacteria promote tumor invasion and metastasis through various pathways [15], and intratumoral microbes influence both tumor diversity and invasive properties [16]. *Fusobacterium nucleatum*, for example, directly drives epithelial–mesenchymal transition, boosting cellular invasion [17]. These observations raise the possibility that microbes may similarly influence endometrial invasion and epithelial plasticity in adenomyosis.

While we understand how microbes drive invasion in cancer and other diseases, their role in adenomyosis remains largely unexplored. Key questions persist: Which specific microorganisms affect endometrial cell behavior? Do they promote classical EMT, an endothelial‐like transition, or a distinct epithelial–endothelial transition in adenomyotic lesions? How do these microbial influences promote ectopic endometrial invasion into the myometrium? Answering these questions could transform our understanding of adenomyosis and open new therapeutic avenues targeting both microbiome and epithelial plasticity.

## 2 Methods

### 2.1 Subject Recruitment and Sample Collection

This study was approved by the Ethics Committee of the Institutional Review Board at the Sixth Affiliated Hospital of Sun Yat‐sen University and conducted in accordance with the Declaration of Helsinki (2020ZSLYEC‐281). Written informed consent was obtained from all participants prior to enrollment. The study cohort comprised eight individuals: five diagnosed with adenomyosis (AM group, mean age 42.3 ± 5.7 years) and three healthy controls (CT group, mean age 39.8 ± 4.2 years). They had not received any hormonal treatment in the three months prior to surgery.

For patients with adenomyosis, all operations were performed by experienced gynecologic surgeons specializing in adenomyosis. Adenomyotic lesions were first identified intraoperatively by inspection and palpation; in women with adenomyosis, the lesions are typically diffuse and macroscopically evident as firm, fibrotic areas within the uterine wall that are readily distinguishable from surrounding normal tissue. From these grossly identified lesion regions, full‐thickness uterine wall blocks were obtained from the uterine corpus. Because adenomyotic lesions are typically diffuse and extensive, there was ample tissue available for both scRNA‐seq and parallel histological validation. Adjacent sections from the same blocks were subjected to H&E staining to confirm the presence of endometrial glands and stroma within the myometrium, consistent with the pathological definition of adenomyosis (Figure S1A). Only blocks containing histologically confirmed adenomyotic lesions were used for subsequent dissociation and scRNA‐seq. For control subjects, full‐thickness uterine tissue (from endometrium to perimetrium) was acquired from hysterectomy procedures.

Fresh tissue samples designated for scRNA‐seq were immediately placed in MACS Tissue Storage Solution (Miltenyi Biotec, #130‐100‐008) and transported on ice for subsequent procedures.

### 2.2 Single‐Cell RNA Sequencing and Data Processing

To generate single‐cell suspensions, fresh tissue samples were subjected to enzymatic digestion for 20 min at 37°C using a cocktail containing 0.35% collagenase IV, 5.2 mg/mL papain, and 120 U/mL DNase I. Following digestion, the suspension was passed through a filter. Erythrocytes and non‐viable cells were subsequently depleted using the MACS Red Blood Cell Lysis Solution and the Miltenyi Dead Cell Removal Kit. The final concentration of viable cells was adjusted to a range of 700 to 1200 cells per microliter.

Single‐cell partitioning was achieved by loading the prepared cell suspensions onto a 10x Genomics Chromium Controller with the Single‐Cell 3' (V3) chemistry. The resulting barcoded libraries were sequenced on an Illumina NovaSeq 6000 platform, aiming for a sequencing depth of approximately 20 000 reads per cell.

The initial processing of raw sequencing files was performed with the Cell Ranger software suite (v.7.0.0). Raw FASTQ files were aligned to the GRCh38 human reference genome, and UMI‐based gene expression matrices were generated for each sample. Unmapped reads were extracted for subsequent microbial analysis. Following alignment, quality control steps were implemented in Seurat (v.4.3.0) [18]. We excluded cells with mitochondrial gene content exceeding 20% or expressing fewer than 300 genes. Potential doublets were computationally identified using DoubletFinder (v.2.0.3) [19] and removed.

### 2.3 Data Integration and Cell Type Identification

To consolidate data from all 8 samples and mitigate batch effects, we employed the Harmony algorithm to integrate the datasets. Gene expression values were normalized using a global scaling factor of 10 000, followed by a log‐transformation. We then identified the top 2000 most variable genes for subsequent dimensionality reduction. Principal component analysis (PCA) was applied, and the top 30 principal components were selected to construct a UMAP for visualization. Cell clustering was achieved using the Leiden algorithm, with various resolution parameters (from 0.4 to 1.4) evaluated to determine the optimal clustering structure.

The biological identity of each cell cluster was determined by examining the expression of canonical marker genes. Major cell types were classified using the following markers: epithelial cells (EPCAM and CDH1), endothelial cells (VWF and PECAM1), smooth muscle cells (SMCs; ACTA2 and MYH11), mesenchymal cells (COL1A1, DCN, PDGFRA, and APOD), myeloid cells (CD68, PTPRC, and CD14), lymphocytes (PTPRC, CD3D, CD3E, and CD2), and uterine natural killer cells (uNK; NCAM1 and TYROBP).

### 2.4 Trajectory Inference and Pseudotime Analysis

Epithelial and endothelial cell populations were extracted and reclustered for trajectory analysis. PAGA (Partition‐based Graph Abstraction) [20] implemented in Scanpy (v1.10.0) [21] was used to infer cellular trajectories and identify intermediate cell states. The analysis was performed using the top 2,000 highly variable genes, with neighbor graph construction using 15 neighbors and UMAP embedding for visualization. Pseudotime analysis was conducted using diffusion maps, and gene expression dynamics along trajectories were analyzed using partition‐based graph abstraction connectivity.

### 2.5 Microbial Identification Using CSI‐Microbes

Bacterial identification from single‐cell RNA sequencing data was performed using the CSI‐Microbes pipeline (https://github.com/ruppinlab/CSI‐Microbes‐identification) [22]. Unmapped reads extracted from the Cell Ranger alignment output were aligned to a comprehensive microbial reference database. This database was constructed by downloading all complete viral, bacterial, and fungal genomes from RefSeq, comprising a total of 4932 genomes. The pipeline utilizes a combination of k‐mer matching and sequence alignment to identify microbial sequences while filtering for potential contamination from the host genome and sequence vectors.

Expected contamination levels were estimated using negative controls and environmental samples processed in parallel. The significance of microbial detection was assessed using a binomial test comparing observed vs. expected read counts, with *p*<0.05 considered statistically significant. Taxonomic classification was performed using Kraken2 (v2.1.2) [23] with a confidence threshold of 0.1.

### 2.6 2bRAD‐M Sequencing for Microbial Validation

FFPE tissue sections (10 µm thickness) were deparaffinized using xylene and rehydrated through graded ethanol series. DNA extraction was performed using the QIAamp DNA FFPE Tissue Kit (Qiagen) according to the manufacturer's protocol. DNA quality and quantity were assessed using NanoDrop spectrophotometry and Qubit fluorometry.

The 2bRAD‐M library preparation was performed following the protocol described by Wang et al. [24] with minor modifications. Approximately 1–200 ng of DNA was digested with 4 U of BcgI (NEB) for 3 h at 37°C. Subsequently, adaptors were ligated to the DNA fragments at 4°C for 12 h in a 20 µl reaction volume containing 10 µl of digested DNA, 0.2 µm of each adaptor, and 800 U of T4 DNA ligase (NEB). The ligation products were then amplified, and the resulting PCR products were separated on an 8% polyacrylamide gel. DNA bands of approximately 100 bp were excised, and the DNA was recovered by diffusion in nuclease‐free water. Sample‐specific barcodes were introduced in a final PCR step using platform‐specific primers. The purified PCR products were sequenced on an Illumina Nova PE150 platform. The 2bRAD‐M service was provided by Qingdao OE Biotech Co., Ltd. (Qingdao, China).

Sequencing data were analyzed using a custom pipeline to ensure accurate species identification and quantification [25, 26]. After quality control, all sequenced 2bRAD tags were mapped against a comprehensive 2bRAD marker database containing tags unique to 86 022 microbial species. To control for false‐positive species identification, a G‐score was calculated for each identified species, with a threshold of G >5 applied. The relative abundance of each species was determined by calculating the ratio of its genomic coverage (average read coverage of all its markers) to the total genomic coverage of all identified species within the sample. Microbial diversity (Chao1 richness index) metrics were calculated using the vegan package in R.

### 2.7 Multiplex Immunofluorescence and FISH

FFPE tissue sections (4 µm thickness) were subjected to antigen retrieval using citrate buffer (pH 6.0) at 95°C for 20 min. Multiplex immunofluorescence staining was performed using the TSAPLus kit (Servicebio, #G1226) according to the manufacturer's protocol. Primary antibodies included: Anti‐pan Cytokeratin (1:250; Abcam, #ab7753), Anti‐VEGFA Mouse mAb (1:250; Servicebio, #GB14165) and Anti‐CD31 Mouse mAb (1:300; Servicebio, #GB12064). Secondary antibodies were HRP‐conjugated.

For FISH detection of *S. paucimobilis*, a specific 16S rRNA probe was designed using the ARB software package. The probe sequence (5′‐GGGCAGATTCCCACGCGT‐3) was labeled with Cy3 fluorophore. FISH was performed using a standard protocol with hybridization at 46°C for 16 h. Nuclei were counterstained with DAPI (1 µg/ml). Slice scanning was performed using Pannoramic 250FLASH (3DHISTECH).

### 2.8 Western Blot Analysis

Protein extraction was performed using RIPA lysis buffer supplemented with protease and phosphatase inhibitors. Protein concentrations were determined using the BCA assay kit (Thermo Fisher). Equal amounts of protein (30 µg) were separated by SDS‐PAGE and transferred to PVDF membranes. Membranes were blocked with 5% non‐fat milk and incubated overnight at 4°C with primary antibodies against EPCAM (1:1000; Servicebio, #GB12274), PECAM1/CD31 (1:1000; Servicebio, #GB153151), MMP2 (1:1000; Servicebio, #GB11130), VEGFA (1:1000; Servicebio, #GB11034B), phospho‐NF‐κB p65 (Ser536) (1:1000; Servicebio, #GB113882), and β‐actin (1:5000; Servicebio, #GB15003) as loading control. After incubation with HRP‐conjugated secondary antibodies, bands were visualized using enhanced chemiluminescence and quantified using ImageJ software. All experiments were performed in triplicate.

### 2.9 Immunofluorescence for NF‐κB p65 Nuclear Translocation

For p65 nuclear translocation analysis in vitro, primary human endometrial epithelial cells were cultured on glass coverslips and subjected to co‐culture with *S. paucimobilis* for 6 h. Cells were fixed with 4% paraformaldehyde, permeabilized with 0.1% Triton X‐100, blocked with 5% BSA, and incubated overnight at 4°C with anti‐NF‐κB p65 antibody (1:200; Abcam, #Ab32536). After washing, cells were incubated with Alexa Fluor 594‐conjugated secondary antibody (1:500; Thermo Fisher) and counterstained with DAPI. Nuclear‐to‐cytoplasmic ratio of p65 fluorescence was quantified using ImageJ software to assess nuclear translocation.

### 2.10 Mouse Adenomyosis Models

Animal experiments were approved by the Institutional Animal Care and Use Committee and conducted in accordance with institutional guidelines. Female CD1 mice (8 weeks old, 20–25 g) and female CD1 newborn mice were obtained from Gnotobio Co., Ltd. (China) and housed in pathogen‐free conditions with 12‐h light/dark cycles and ad libitum access to food and water.

Three experimental groups were established (*n* = 5 per group): (1) *S. paucimobilis* (#JCM7518, Japan Collection of Microorganism; isolated from Vaginal swab) inoculation group; (2) tamoxifen‐induced adenomyosis group (positive control); (3) PBS vehicle control group. For bacterial inoculation, S. paucimobilis was cultured in brain heart infusion agar at 30°C for 18 h. Bacteria were harvested, washed with sterile PBS, and resuspended to a concentration of 1 × 10^8^ CFU/ml. Mice were anesthetized with isoflurane, and 50 µl of bacterial suspension was instilled into the vagina using a sterile pipette tip. Control mice received equivalent volumes of PBS. The procedure was repeated once every two days for two weeks. For the tamoxifen model, neonatal mice were orally administered tamoxifen (1 mg/kg; Sigma, T5648) suspended in a peanut oil/lecithin/condensed milk mixture (2:0.2:3, v/v) on days 2 through 5 after birth, at a dose volume of 5 µL/g body weight [27].

Mice were monitored daily for signs of distress and euthanized 4 weeks post‐treatment using CO_2_ asphyxiation followed by cervical dislocation. Uterine tissues were collected for histological and molecular analyses.

### 2.11 In Vitro Co‐Culture Experiments

Primary human endometrial epithelial cells were provided by Wuhan Warner Bio Technology Co., Ltd (#WN‐261406). Subculture was performed using companion epithelial cell culture medium (#WN‐1001S). Co‐culture experiments were performed at a multiplicity of infection (MOI) of 10:1 (bacteria:cells; 10e6 cfu/ml,100 ul: 10e4/Samp). Time‐course experiments were conducted with sample collection at 12 (T12), and 24 (T24) h post‐infection. At each time point, cells were washed with PBS to remove non‐adherent bacteria, and RNA was extracted for transcriptome analysis. Control wells (T0) without bacteria were processed in parallel.

### 2.12 In Vitro Inhibitor Experiment Design

To investigate the functional roles of NF‐κB and MMP signaling in ^*^S. paucimobilis^*^‐induced EET, we designed a 4‐group experiment using primary human endometrial epithelial cells: Ctrl: Vehicle control (culture medium only); Live: Live *S. paucimobilis* co‐culture (MOI 10:1); Live + NF‐κB inh: *S. paucimobilis* co‐culture + Bay 11–7082 (10 µm; Sigma, #B5556), an NF‐κB inhibitor that blocks IκBα phosphorylation; Live + MMP inh: *S. paucimobilis* co‐culture + GM6001 (25 µm; Sigma, #CC1010), a broad‐spectrum MMP inhibitor.

Inhibitors were added 1 h prior to bacterial co‐culture and maintained throughout the experiment. Cells were harvested at 48 h post‐co‐culture for Western blot analysis. For p65 nuclear translocation studies, cells were fixed at 6 h post‐co‐culture for immunofluorescence analysis.

### 2.13 In Vivo Inhibitor Experiment Design

To systematically validate the TNFα → NF‐κB → MMP/ECM signaling cascade in vivo, we expanded the mouse model to include a comprehensive 6‐group experiment (*n* = 5 per group): Ctrl: Vehicle (PBS) vaginal inoculation; Live: Live *S. paucimobilis* (10^7^ CFU) vaginal inoculation; Sup: Bacterial culture supernatant (sterile‐filtered conditioned medium containing secreted metabolites); Live + NF‐κB inh: *S. paucimobilis* + Bay 11–7082 (5 mg/kg, i.p., every other day); Live + αTNF: *S. paucimobilis* + Infliximab (10 mg/kg, i.p., twice weekly); Live + MMP inh: *S. paucimobilis* + GM6001 (100 mg/kg, i.p., daily)

All inhibitors were administered starting from day 1 of bacterial inoculation and continued throughout the 4‐week experimental period. Mice were euthanized 4 weeks post‐treatment, and uterine tissues were collected for immunofluorescence analysis.

### 2.14 Scanning Electron Microscopy (SEM) Analysis

To observe bacterial–epithelial cell interactions, in vitro co‐culture samples were fixed with 2.5% glutaraldehyde for 2 h, washed 3 times with 0.1 m phosphate buffer (pH 7.4), and post‐fixed in 1% osmium tetroxide for 1 h. The samples were then dehydrated by a fractionated ethanol series and chemically dried using hexamethyldisilazane (HMDS). The dried sample was mounted on an aluminum short, sputtered with 8–10 nm gold, and examined under a field emission scanning electron microscope (SU8100, HITACHI) at an accelerating voltage of 3–15 kV. Capture at least 5 different fields of view for each sample at low (500–1000×) and high (5000–20000×) magnifications.

### 2.15 RNA Extraction and Bulk RNA Sequencing

Total RNA was extracted using the RNeasy Mini Kit (Qiagen) according to the manufacturer's protocol. RNA quality and quantity were assessed using the Agilent 2100 Bioanalyzer, and only samples with RNA integrity numbers (RIN) ≥7.0 were used for sequencing.

RNA sequencing libraries were prepared using the TruSeq Stranded mRNA Library Prep Kit (Illumina) with polyA selection for mRNA enrichment. Libraries were sequenced on an Illumina NovaSeq 6000 platform with paired‐end 150 bp reads.

### 2.16 Statistical Analysis

All statistical analyses were performed using R (v4.2.0) and GraphPad Prism (v9.0). Data preprocessing included log‐transformation for gene expression data and evaluation of outliers using the interquartile range (IQR) method. Continuous variables are presented as mean ± standard deviation (SD) or median with interquartile range, depending on data distribution. Sample sizes (n) are specified in each figure legend.

For comparisons between two groups, Student's two‐tailed t‐test was used for normally distributed data (assessed by the Shapiro–Wilk test, *p*>0.05), while the Mann–Whitney U test was applied for non‐normally distributed data. For comparisons among multiple groups, one‐way ANOVA followed by Tukey's post‐hoc test was used for normally distributed data, and Kruskal–Wallis test followed by Dunn's post‐hoc test was used otherwise. Categorical variables were compared using Fisher's exact test. Multiple comparisons were adjusted using the Benjamini‐Hochberg false discovery rate (FDR) correction. Statistical significance was defined as two‐tailed *p*<0.05. All experiments were performed with at least three biological replicates unless otherwise stated.

## 3 Results

### 3.1 Overview of Single‐Cell Transcriptomic Profiles in Healthy and Adenomyosis Human Uterine Tissues

**FIGURE 1:**
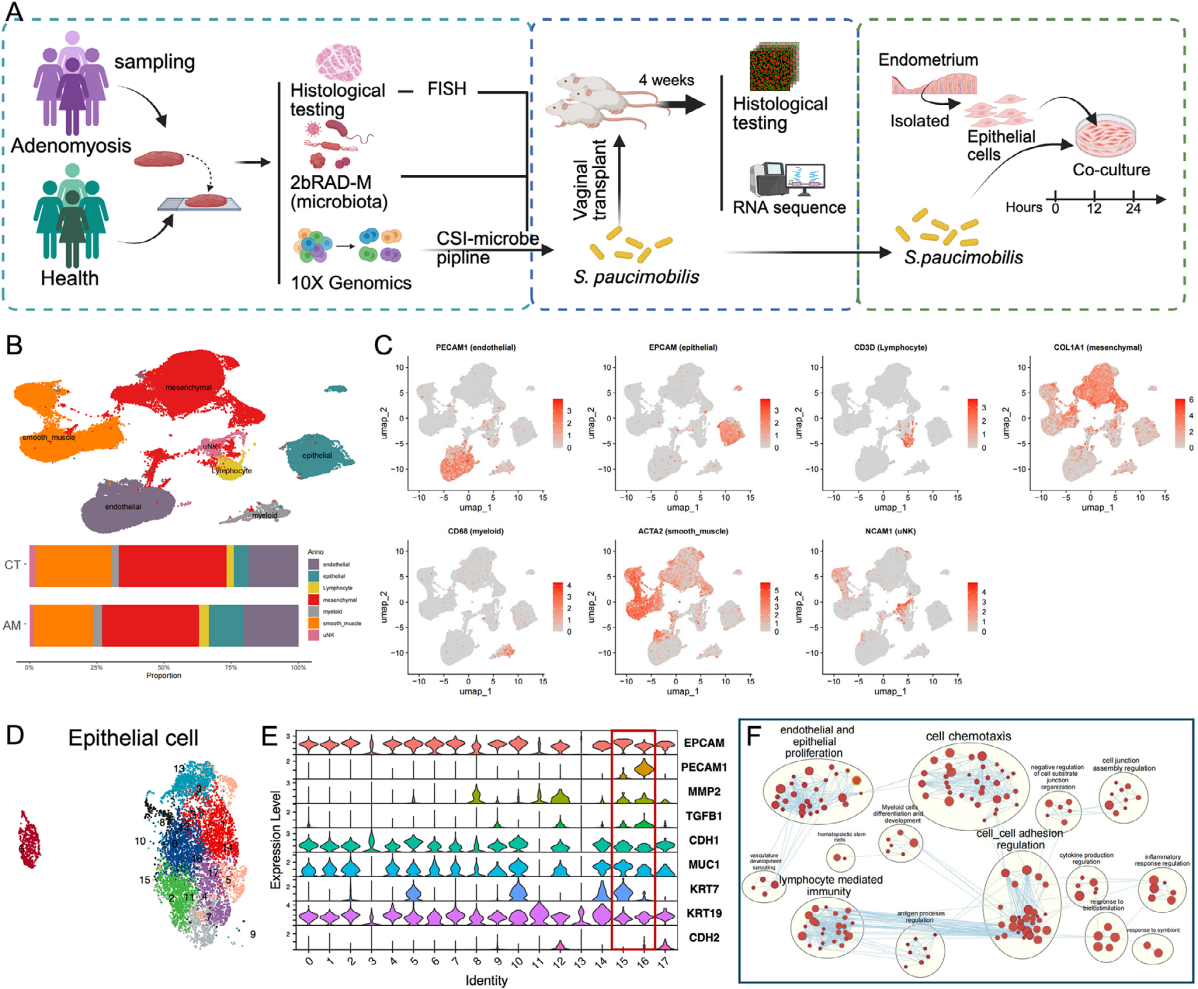
Overview of single‐cell transcriptome analysis and identification of epithelial–endothelial transition in adenomyosis. (A). Comprehensive experimental flowchart of the study; (B). UMAP visualization based on cell type‐specific marker gene expression showing 7 major cell populations: epithelial cells, endothelial cells, smooth muscle cells, mesenchymal cells, myeloid cells, lymphocytes and uterine natural killer cells. Stacked plots showed significantly higher proportions of epithelial and endothelial cells in the AM group than in the CT group; (C). Heatmap of typical marker gene expression of each cell type; (D). Reclustering analysis of epithelial cell subpopulations; (E). Violin plot of characteristic gene expression showing that clusters C15 and C16 express both the epithelial marker EPCAM and the endothelial marker PECAM1, indicating that epithelial–endothelial transformation (EET) occurs in these cells; (F). Functional enrichment analysis of EET cells (C15 and C16) showed significant enrichment for biological processes such as endothelial and epithelial proliferation, cell chemotaxis and regulation of cell–cell adhesion.

The comprehensive experimental pipeline for this study is illustrated in Figure 1A. We recruited a total of 8 subjects, comprising 5 patients with adenomyosis (AM group; Not receiving GnRHa treatment) and 3 healthy women (CT group; Surgery for multiple uterine fibroids). Single‐cell transcriptome sequencing was performed using the 10X Genomics platform to profile cells from these 8 donors (Figure S1A). Following stringent quality control procedures, we obtained 87 236 high‐quality cells, with a median of 2871 genes and 7457 unique transcripts per cell (Figure S1B–E). The Harmony method was employed to integrate data from individual samples (Figure S1E).

Based on expression of established cell‐type‐specific markers, we identified seven major cell populations (Figure 1B,C). Notably, we observed a significantly higher proportion of epithelial cells and endothelial cells in the adenomyosis group compared to the control group (Figure 1B), suggesting enhanced cellular proliferation and angiogenesis in adenomyotic lesions.

### 3.2 Identification of Epithelial–Endothelial Transition in Adenomyosis Lesions

To investigate cellular transformation mechanisms in adenomyosis, we extracted epithelial cells for detailed analysis (Figure 1C). Remarkably, we discovered that two distinct epithelial cell clusters (C15 and C16) simultaneously expressed both the endothelial cell marker PECAM1 and the epithelial cell marker EPCAM (Figure 1E), indicating a hybrid cellular phenotype. Based on this unique co‐expression pattern, we characterized these cells as having undergone epithelial–endothelial transition (EET), a previously described cellular plasticity process in adenomyosis [14]. Functional enrichment analysis revealed that clusters C15 and C16 were enriched for biological processes including endothelial and epithelial proliferation, cell chemotaxis, and cell–cell adhesion regulation (Figure 1F).

**FIGURE 2:**
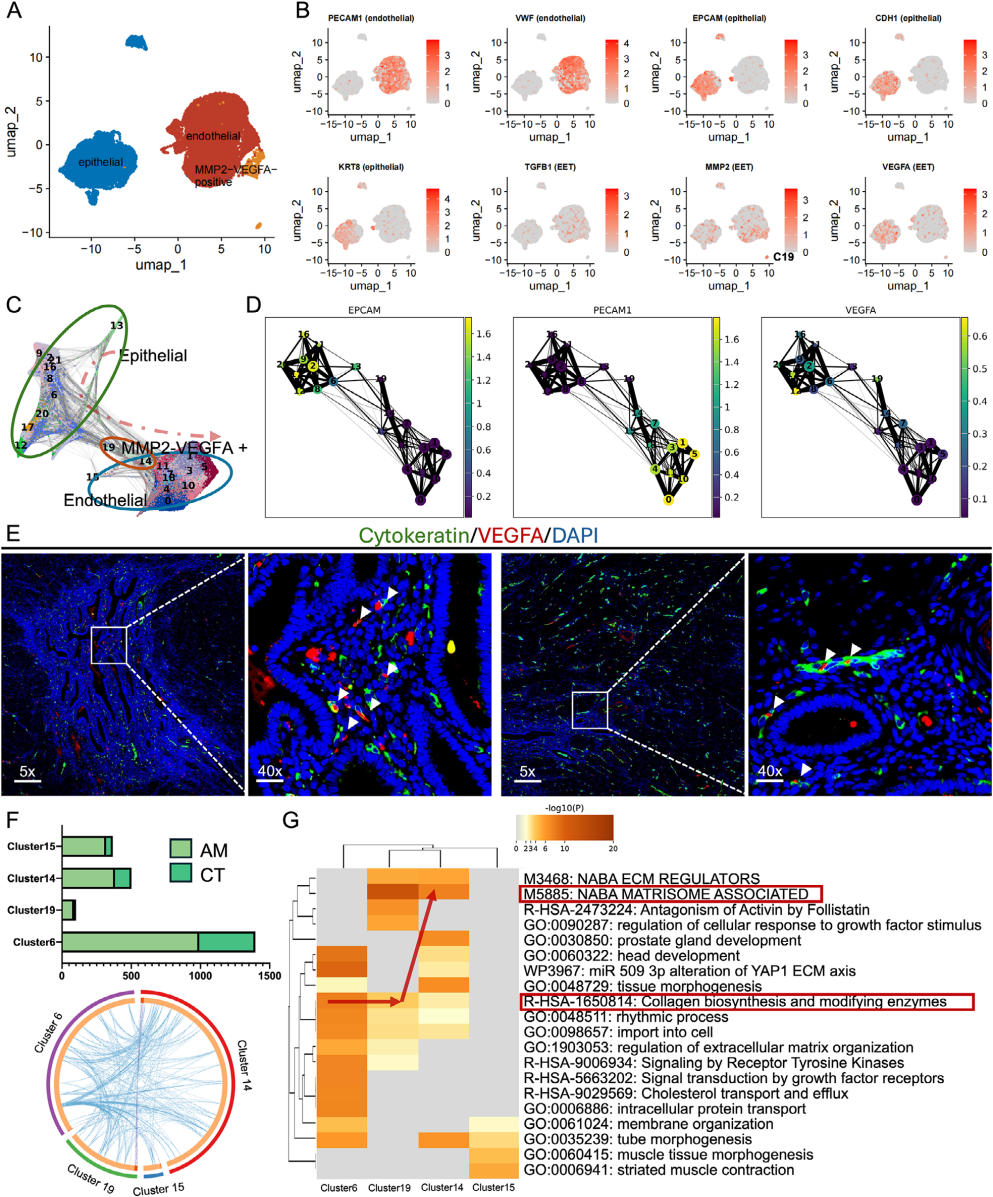
Trajectory analysis and molecular characterization of the epithelial–endothelial transformation process. (A). UMAP plot of the joint reclustering analysis of epithelial and endothelial cells; (B). Heatmap of the expression of key transformation‐related genes (EPCAM, CDH1, KRT8, PECAM1, VWF, TGFB1, MMP2, VEGFA) in different cell clusters. MMP2 was highly expressed in cluster C19; (C). PAGA trajectory inference analysis identified “intermediate cell” populations (C19 and C14) located between epithelial and endothelial cell states; (D). Expression patterns of the transformation‐related genes MMP2 and VEGFA in the trajectory analysis, showing high expression in the intermediate state cells; (E). Multiplex immunofluorescence validation in full‐thickness uterine wall sections from patients with histologically confirmed adenomyosis showed the presence of cells expressing both epithelial and endothelial markers, confirming the findings of the computational analysis; (F). Differences in the distribution of intermediate state cells (C19, C14) and their connectivity clusters (C6, C15) in the AM and CT groups and analysis of the strength of intercellular connections; (G). Functional enrichment analysis of the C6→C19→C14 trajectory showed that the collagen biosynthesis process was active in C6 and gradually weakened in C19 and C14, while the NABA matrix‐related process was enhanced from C19.

Further, epithelial cells and endothelial cells were extracted and reclustered (Figure 2A; Figure S1F). Using established markers to distinguish epithelial cells (EPCAM, CDH1, and KRT8) from endothelial cells (PECAM1 and VWF), we also examined the expression of key transformation‐related genes including TGFB1, MMP2, and VEGFA (Figure 2B). Notably, MMP2, a critical mediator of epithelial transformation and extracellular matrix remodeling, was highly expressed in cluster 19 cells (Figure 2B).

Employing PAGA trajectory inference analysis, we identified a distinct population of “intermediate cells” positioned between epithelial and endothelial cell states, comprising clusters C19 and C14, both characterized by high expression of MMP2 and VEGFA (Figure 2C,D). To validate our computational findings, we performed multiplex immunofluorescence staining on full‐thickness uterine wall sections from patients with histologically confirmed adenomyosis and confirmed the presence of cells co‐expressing epithelial and endothelial markers (Figure 2E). Focusing on the intermediate state cells (C19, C14) and their connected clusters (C6, C15), we observed that these cell populations were predominantly distributed in the adenomyosis group and exhibited strong intercellular connectivity (Figure 2F). Functional enrichment analysis of the C6→C19→C14 trajectory revealed dynamic changes in cellular programs: collagen biosynthesis and modifying enzyme processes were highly active in C6 cells and progressively diminished in C19 and C14, while NABA matrisome‐associated processes became increasingly active from C19 onward (Figure 2G). These findings collectively demonstrate the occurrence of epithelial–endothelial transition as a key pathological mechanism in adenomyosis lesions.

### 3.3 Cell–Cell Communication Networks Revealed Signaling Mechanisms Underlying Epithelial–Endothelial Transition

To further dissect the molecular mechanisms underlying EET, we performed comprehensive cell–cell communication analysis to characterize intercellular signaling networks among epithelial, endothelial, and transitional cell populations. The global communication network revealed extensive signaling interactions between these three cell types, with transitional cells serving as critical mediators connecting epithelial and endothelial compartments (Figure S2A). Analysis of incoming and outgoing signaling patterns demonstrated distinct communication profiles: epithelial cells primarily functioned as signal senders, endothelial cells served as both senders and receivers, while transitional cells exhibited the highest signal reception capacity (Figure S2B).

Pathway‐specific analysis identified several keys signaling networks driving EET. The VEGF signaling pathway showed the strongest activity among all analyzed pathways, with predominant signaling from transitional to endothelial cells (Figure S2C,D). UMAP visualization of VEGF pathway components (VEGFA, VEGFB, VEGFC, KDR) confirmed their preferential expression in transitional and endothelial cell populations, supporting the critical role of VEGF‐mediated angiogenic signaling in EET progression. The WNT signaling network exhibited complex bidirectional communication patterns, with WNT2 showing specific expression in transitional cells, indicating its potential role in cell fate determination during epithelial–endothelial transformation (Figure S2E).

Additionally, PDGF and FGF signaling pathways demonstrated robust activity primarily in epithelial and transitional cell populations (Figure S2F,G). The PDGF pathway showed strong autocrine and paracrine signaling within epithelial cells, potentially regulating cellular proliferation and migration during early stages of transformation. FGF signaling exhibited widespread distribution across all cell types, suggesting its role in maintaining cellular plasticity and supporting the dynamic nature of EET. These comprehensive cell–cell communication analyses reveal that EET progression is orchestrated through coordinated activation of multiple signaling networks, with transitional cells functioning as central nodes integrating diverse molecular signals to drive epithelial–endothelial transformation.

### 3.4 *Sphingomonas paucimobilis* Induced Epithelial–Endothelial Transition in Adenomyosis Lesions

**FIGURE 3:**
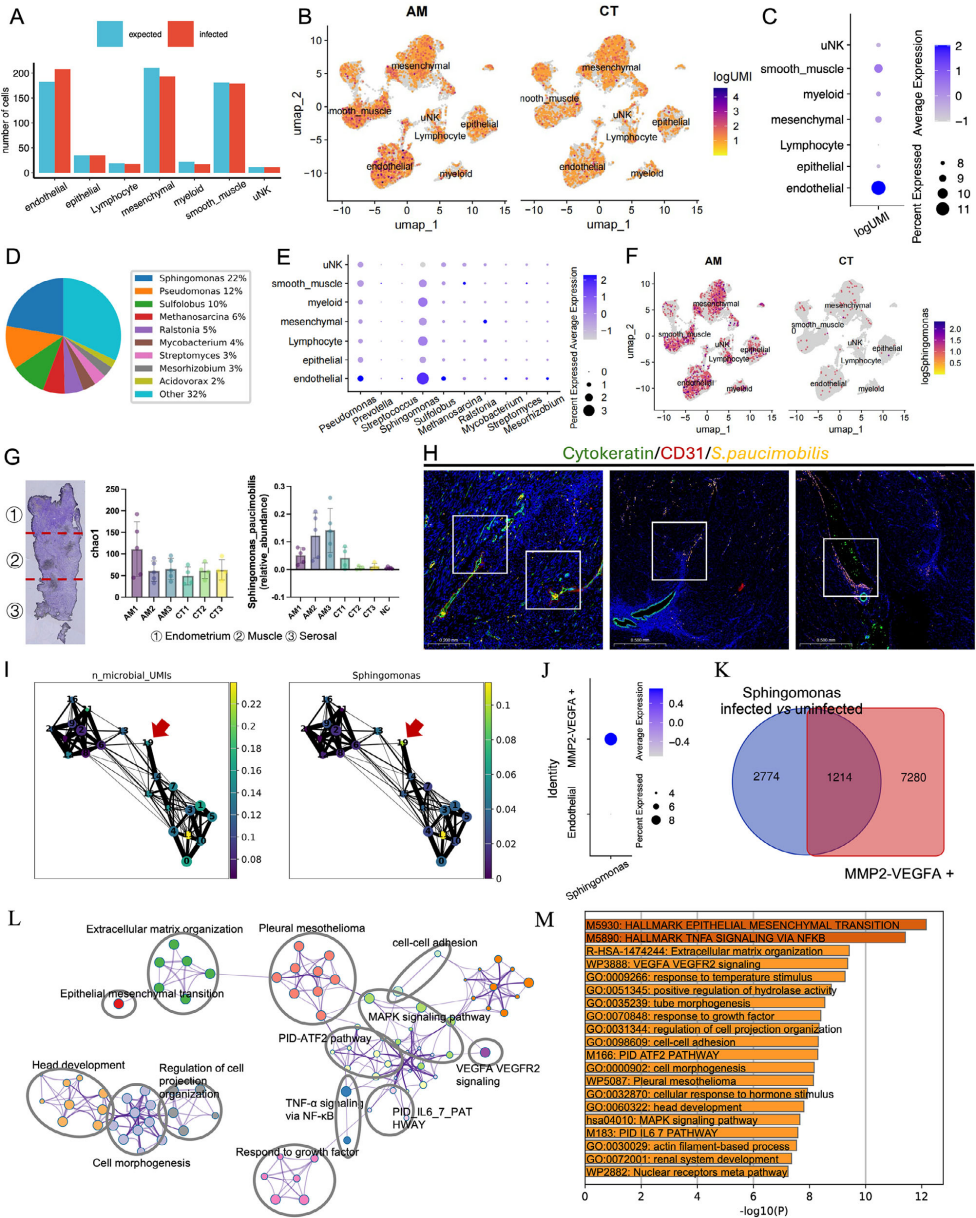
Identification and localization of oligotrophic *Sphingomonas paucimobilis* in adenomyosis foci. (A–C). CSI‐Microbes analysis procedure and results showing that the number of bacterial sequences detected in endothelial cells significantly exceeded the baseline of expected contamination levels, and a moderate number of bacterial sequences were also detected in epithelial cells; (D–E). Bacterial Taxonomic Annotation Results Showing *Sphingomonas spp*. As the Dominant Bacterial Group, Mainly Localized to Endothelial Cells. (F). UMAP visualization showed that *Sphingomonas spp*. were widely distributed in all cell types in the AM group, while they were almost absent in the CT group. (G). 2bRAD‐M probe‐based detection showed significantly higher microbial Chao1 diversity index in the endometrium and superficial myometrium of the AM group compared with controls, with no significant differences in the serosal layer. *S. paucimobilis* signals were enriched in adenomyosis uteri compared with controls, in which only low‐level signals were detected in the endometrium; (H). Multiplex immunofluorescence combined with S. paucimobilis‐specific FISH probes showed bacterial aggregation at the leading edge of adenomyosis lesions and migratory trajectories that co‐localized directly with cells undergoing EET; (I,J). Microbial information integration analysis revealed a high abundance of microbial sequences and *Sphingomonas*‐specific labels preferentially detected in transformed state cells (C19 and C14; MMP2 and VEGFA positive); (K). Comparative gene expression analysis identified 1214 genes that were differentially expressed in both *Sphingomonas*‐infected cells and MMP2/VEGFA‐positive cell clusters; (L‐M). pathway enrichment analysis revealed that these 1214 genes were significantly enriched in epithelial cell transformation, TNFα/NF‐κB signaling, extracellular matrix organization, and VEGFA‐VEGFR2 signaling pathways.

The CSI‐Microbes pipeline was employed for bacterial identification of adenomyosis lesions. Analysis revealed that the number of bacterial sequences detected in endothelial cells significantly exceeded the expected baseline contamination levels, while a moderate number of bacterial sequences were also detected in epithelial cells (Figure 3A–C, detailed in Materials and Methods). Taxonomic annotation of the detected bacterial fragments identified *Sphingomonas spp*. as the predominant bacterial genus, with preferential localization to endothelial cells (Figure 3D,E). UMAP visualization demonstrated that *Sphingomonas spp*. was widely distributed across all cell types in the adenomyosis group but was virtually absent in the control group (Figure 3F).

To validate the authenticity of these bacterial signals and exclude potential contamination artifacts, we performed layer‐specific microbiological analysis of uterine FFPE (formalin‐fixed paraffin‐embedded) samples using the 2bRAD‐M probe‐based detection assay (Figure 3G). Tissue samples were sectioned into three anatomical layers: endometrium, myometrium, and serosa, with blank paraffin sections serving as negative controls to monitor for contamination. The microbial Chao1 diversity index was significantly elevated in endometrial and superficial myometrial tissues in the adenomyosis group compared to controls, with no significant differences observed in the serosal layer (Figure 3G). In addition, 2bRAD‐M quantification revealed higher *Sphingomonas paucimobilis (S. paucimobilis)* signals in adenomyosis uteri than in controls (Figure 3G). Spatial localization studies using multiplex immunofluorescence combined with *S. paucimobilis*‐specific FISH probes on paraffin‐embedded sections further confirmed the presence of this bacterium within the endometrium and myometrium of adenomyosis lesions, where bacteria clustered at the leading edges and migratory tracks of adenomyotic foci and directly co‐localized with cells undergoing EET (Figure 3H). These orthogonal validations strongly support the authenticity of the microbial signals identified by CSI‐Microbes and the enrichment of *S. paucimobilis* in adenomyosis.

Integrative analysis mapping microbial information onto our single‐cell dataset revealed that high abundances of microbial sequences and *Sphingomonas*‐specific tags were preferentially detected in transition‐state cells (clusters C19 and C14; MMP2 and VEGFA^+^) (Figure 3I,J). Comparative gene expression analysis identified 1,214 genes that were differentially expressed in both *Sphingomonas*‐infected cells and MMP2 and VEGFA^+^ cell clusters, representing the molecular signature of *Sphingomonas*‐induced cellular transformation (Figure 3K). Pathway enrichment analysis demonstrated that these 1,214 genes were significantly enriched in epithelial cell transformation, TNFα signaling via NF‐κB, extracellular matrix organization, and VEGFA‐VEGFR2 signaling pathways (Figure 3L,M), establishing the molecular mechanisms by which *S. paucimobilis* drived EET in adenomyosis.

### 3.5 *S. paucimobilis* Infection Induces Adenomyosis Development in Mouse Models

**FIGURE 4:**
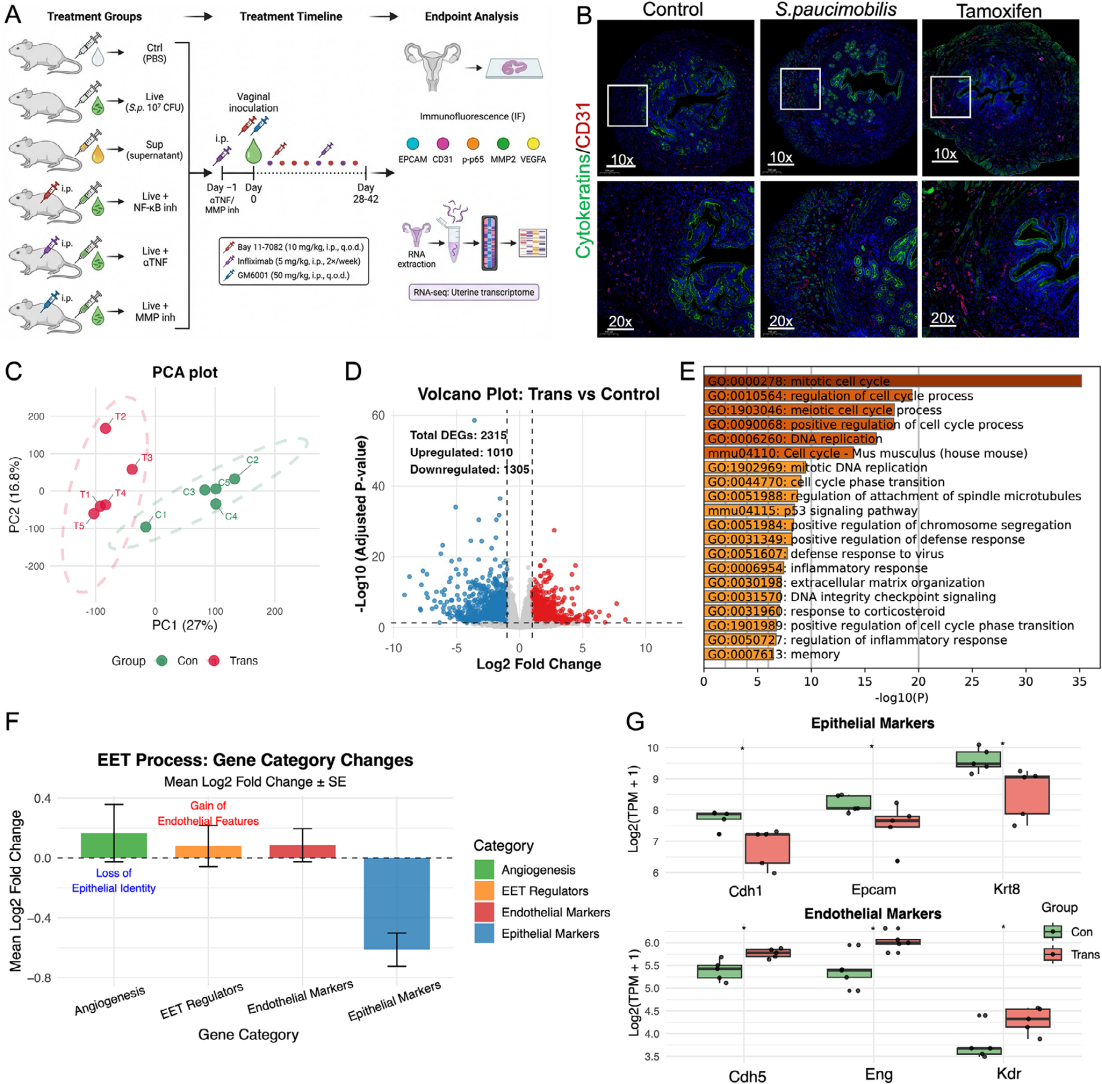
*S. paucimobilis* infection‐induced adenomyosis model in mice. (A). Schematic diagram of the 6‐group in vivo experiment design; (B). Immunofluorescence analyses showed that *S. paucimobilis* vaginal inoculation successfully induced the development of adenomyosis in the uterus of adult CD1 mice, with deep infiltration of ectopic endometrial glands and mesenchyme into the myometrium. When compared with the tamoxifen‐induced model, the *S. paucimobilis* group exhibited adenomyosis lesions with histopathological features comparable to those observed in the classical model; (C). Principal component analysis of genome‐wide expression profiles of *S. paucimobilis* after inoculation showed drastic changes in gene expression; (D). Volcano plot of differentially expressed genes (DEGs) showed that 2315 differentially expressed genes were identified (|log2FC| ≥1, adjusted *p*<0.05); (E). Functional enrichment analysis showed significant correlation with cell mitosis, DNA replication and cell cycle regulation, indicating enhanced cell proliferation after bacterial infection; (F). Expression of genes related to EET regulation, endothelial cell identity and angiogenesis was upregulated; (G). Epithelial cell marker genes (Epcam, Krt8, Krt9, Cdh1, Ocln) were significantly down‐regulated in the *S. paucimobilis* group. ^*^ *p*<0.05, ^**^ *p*<0.01, ^***^ *p*<0.001 vs. Ctrl group.

To establish a causal relationship between *S. paucimobilis* infection and adenomyosis development and to functionally validate the underlying signaling pathways, we designed a comprehensive 6‐group mouse experiment: PBS vehicle control (Ctrl), live *S. paucimobilis* vaginal inoculation (Live), bacterial culture supernatant (Sup), and three inhibitor groups—Live + NF‐κB inhibitor (Bay 11–7082), Live + TNFα‐neutralizing antibody (Infliximab), and Live + MMP inhibitor (GM6001) (Figure 4A). The Sup group, receiving sterile‐filtered conditioned medium containing secreted bacterial metabolites but no viable bacteria, was designed to distinguish between contact‐dependent and metabolite‐mediated effects.

Histological and immunofluorescence analyses demonstrated that vaginal inoculation with *S. paucimobilis* successfully induced adenomyosis development in adult CD1 mice, with ectopic endometrial glands and stroma infiltrating deep into the myometrium (Figure 4B). When benchmarked against the neonatal tamoxifen‐induced model (positive control), the *S. paucimobilis*–inoculated mice exhibited adenomyotic lesions with histopathological features comparable to those observed in the classical tamoxifen model, indicating that bacterial exposure alone is sufficient to drive the development of adenomyosis‐like lesions in the adult uterus (Figure 4B). Critically, the Sup group showed no significant adenomyosis‐like changes—uterine architecture, EPCAM expression, and all other EET‐related markers remained at baseline levels comparable to the Ctrl group (Figure S3). This finding indicates that secreted bacterial metabolites alone are insufficient to induce EET and adenomyosis development; rather, viable *S. paucimobilis* bacteria are required, suggesting a role for active bacterial colonization or contact‐dependent mechanisms.

The global gene expression profile was dramatically altered following *S. paucimobilis* inoculation (Figure 4C), with 2315 differentially expressed genes (DEGs) identified (Figure 4D). Functional enrichment analysis revealed that upregulated DEGs were significantly associated with cell mitosis, DNA replication, and cell cycle regulation, indicating enhanced cellular proliferation in response to bacterial infection (Figure 4E). Categorization of DEGs based on cellular function revealed distinct expression patterns that recapitulated our human findings. Epithelial cell markers, including Epcam, Krt8, Krt9, Cdh1, and Ocln, were significantly downregulated in the *S. paucimobilis *group compared to controls (Figure 4G). Conversely, genes associated with EET regulation, endothelial cell identity, and angiogenesis were predominantly upregulated (Figure 4F; Figure S4). These transcriptomic changes mirror the molecular signature of EET observed in human adenomyosis tissues, providing strong evidence that *S. paucimobilis* infection drives adenomyosis pathogenesis through induction of epithelial–endothelial transition in vivo.

Immunofluorescence of EET‐related protein markers in uterine tissue sections revealed striking morphological differences between experimental groups (Figure S3), consistent with the transcriptomic changes observed in Figure 4D–G. The Live group exhibited the EET signature: significantly decreased EPCAM (epithelial marker), elevated CD31/PECAM1 (endothelial marker), and increased MMP2, VEGFA, and phosphorylated NF‐κB p65 (p‐p65), accompanied by adenomyosis‐like lesion formation. In contrast, all three inhibitor groups showed significant rescue effects: both TNFα‐neutralizing antibody (Infliximab) and NF‐κB inhibitor (Bay 11–7082) treatment markedly reduced p‐p65 levels along with decreased MMP2 and VEGFA expression, confirming that TNFα acts upstream of NF‐κB activation. The MMP inhibitor (GM6001) group showed preserved p‐p65 activation but significantly reduced MMP2 activity and downstream EET markers, confirming that MMPs function as effectors downstream of NF‐κB signaling. All inhibitor groups showed partial restoration of EPCAM expression and reduced lesion formation. These results provide direct in vivo evidence for a linear TNFα → NF‐κB → MMP/ECM signaling cascade driving *S. paucimobilis*‐induced EET.

### 3.6 *S. paucimobilis* Directly Induces Epithelial–Endothelial Transition In Vitro

**FIGURE 5:**
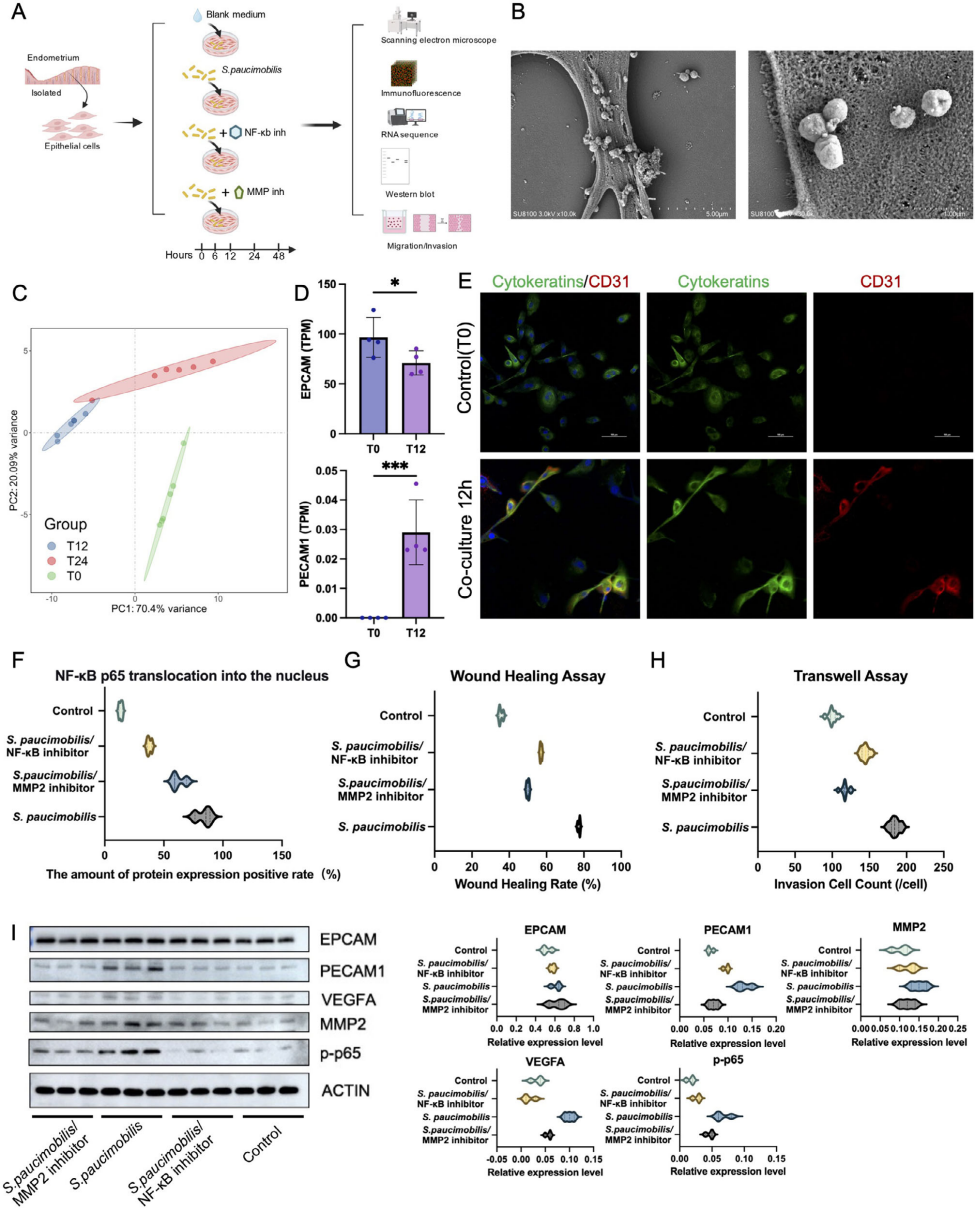
*S. paucimobilis* directly induces epithelial–endothelial transformation in vitro through NF‐κB signaling. (A). Schematic diagram of the 4‐group in vitro experimental design; (B). Scanning electron microscopy analysis confirms the direct physical interaction of *S. paucimobilis* with epithelial cells, with the bacteria adhering to and invading the surface of epithelial cells within 12 h of co‐culture. Scale bar: 5 µm (low magnification), 1 µm (high magnification); (C). Principal component analysis shows dramatic changes in genome‐wide expression patterns, with the most significant change occurring after 12 h of infection (T12). Prolonged co‐cultivation beyond 12 h induces a small number of transcriptome changes; (D). Abundance analysis of cell type‐specific markers confirms progressive loss of epithelial identity and gain of endothelial features: decreased EPCAM expression (*p*<0.001) and increased PECAM1 expression (*p*<0.001); (E). Representative immunofluorescence images of Cytokeratins (green) and CD31 (red) at 0 h (Control) and 12 h post‐co‐culture. After 12 h, epithelial cells express strong CD31 signals while maintaining Cytokeratins expression, indicating the emergence of a hybrid epithelial–endothelial phenotype. Scale bar: 50 µm; (F). Violin plot showing p65 nuclear translocation rate (nuclear/cytoplasmic ratio) at 6 h post‐co‐culture across the 4 groups. The Live group shows robust nuclear p65 accumulation, which is blocked by NF‐κB inhibitor treatment (^***^ *p*<0.001); (G). Violin plot showing wound healing/scratch assay results (percentage wound closure at 24 h) across the 4 groups. *S. paucimobilis* co‐culture significantly enhances wound closure, which is attenuated by both inhibitors (^*^ *p*<0.05, ^**^ *p*<0.01); (H). Violin plot showing Transwell migration assay results (number of migrated cells) across the 4 groups. The Live group shows markedly increased migration, which is reduced by both NF‐κB and MMP inhibition (^**^ *p*<0.01, ^***^ *p*<0.001); (I). Western blot analysis and quantification of EET‐related proteins (EPCAM, PECAM1/CD31, MMP2, VEGFA, p‐p65) at 48 h post‐co‐culture across the 4 groups. The Live group shows decreased EPCAM and increased PECAM1, MMP2, VEGFA, and p‐p65. Both inhibitor groups show significant attenuation of these protein‐level changes, validating the NF‐κB → MMP signaling axis. β‐actin serves as a loading control. ^*^ *p*<0.05, ^**^ *p*<0.01, ^***^ *p*<0.001. Western Blot and Functional Validation of EET In Vitro.

To demonstrate the direct causal relationship between *S. paucimobilis *and EET induction, and to functionally validate the underlying signaling pathways, we established a controlled co‐culture system using primary human endometrial epithelial cells and live *S. paucimobilis* bacteria. The experimental design included a comprehensive 4‐group comparison: vehicle control (Ctrl), live *S. paucimobilis* co‐culture (Live), Live + NF‐κB inhibitor (Bay 11–7082, 10 µm), and Live + MMP inhibitor (GM6001, 25 µm), with inhibitors added 1 h prior to bacterial exposure (Figure 5A).

Scanning electron microscopy analysis confirmed direct physical interaction between *S. paucimobilis *and epithelial cells, with bacteria adhering to and invading the epithelial cell surface within 12 h of co‐culture (Figure 5B). Principal component analysis (PCA) of transcriptomic data demonstrated dramatic shifts in global gene expression patterns, with the most pronounced changes occurring at 12 h post‐infection (T12); notably, extending co‐culture time beyond 12 h did not induce additional significant transcriptomic changes (Figure 5C). Quantitative analysis of cell‐type‐specific markers at the transcriptome level confirmed the progressive loss of epithelial identity and acquisition of endothelial characteristics: EPCAM expression significantly decreased while PECAM1 expression markedly increased in the Live group compared to controls (Figure 5D). Comprehensive marker analysis revealed the temporal progression of cellular transformation, with epithelial markers (EPCAM, CDH1, KRT8) progressively downregulated while endothelial markers (PECAM1, VWF, CD31) and EET‐associated genes (MMP2, VEGFA, SNAI1) were simultaneously upregulated (Figure S5A). Mfuzz clustering analysis grouped genes based on their temporal expression patterns, revealing early‐response genes (rapidly induced within 12 h) and late‐response genes (peak expression at 24 h) (Figures S5B and S6). Immunofluorescence analysis at 12 h post‐co‐culture revealed that epithelial cells in the Live group began expressing strong CD31 signals while maintaining Cytokeratins expression, indicating the emergence of hybrid epithelial–endothelial phenotypes (Figure 5E).

To confirm NF‐κB pathway activation, we assessed p65 nuclear translocation at 6 h post‐co‐culture. Quantitative image analysis revealed that the Live group showed a significantly elevated p65 nuclear translocation rate (83.73 ± 6.62% vs. 13.58 ± 1.49% in Ctrl; *p*<0.001; Figure 5F and Figure S7), indicating robust NF‐κB activation. This nuclear translocation was significantly blocked by NF‐κB inhibitor (Bay 11–7082) treatment (54.42 ± 10.09%; *p*<0.001 vs. Live), while MMP inhibitor treatment did not significantly affect p65 nuclear localization (62.49 ± 5.81%), providing direct evidence that *S. paucimobilis* activates the NF‐κB signaling pathway early in the EET process.

Functional validation through wound healing scratch assays demonstrated that *S. paucimobilis* co‐culture significantly enhanced wound closure rate (77.5 ± 0.6% vs. 50.0 ± 0.7% in Ctrl at 24 h; *p*<0.001; Figure 5G; Figure S8A). Similarly, Transwell migration assays showed markedly increased cell migration in the Live group (184.4 ± 6.6 cells/field vs. 117.6 ± 5.2 in Ctrl; *p*<0.001; Figure 5H; Figure S8B). NF‐κB inhibitor treatment partially attenuated wound closure (57.0 ± 0.5%) and migration (144.6 ± 5.3 cells/field), while MMP inhibitor treatment showed the strongest inhibitory effect on both wound closure (35.5 ± 1.1%) and migration (100.0 ± 5.4 cells/field) (all *p*<0.01 vs. Live), indicating that the NF‐κB pathway and MMP activity are functionally required for *S. paucimobilis*‐induced cellular migration.

To provide protein‐level evidence of EET, we performed Western blot analysis at 48 h post‐co‐culture across the 4 experimental groups (Figure 5I). The Live group showed significantly decreased EPCAM (epithelial marker) and increased PECAM1/CD31 (endothelial marker), confirming epithelial‐to‐endothelial transition at the protein level. MMP2 and VEGFA protein levels were also elevated in the Live group, consistent with enhanced ECM remodeling and angiogenic signaling. Importantly, phosphorylated NF‐κB p65 (p‐p65) was markedly increased, confirming activation of the NF‐κB pathway. Both inhibitor groups showed significant attenuation of these protein‐level changes: NF‐κB inhibition reduced p‐p65 levels and partially reversed all downstream markers (MMP2, VEGFA, PECAM1) while preserving EPCAM expression; MMP inhibition did not affect p‐p65 but significantly reduced MMP2 activity and the downstream EET phenotype. These results provide mechanistic validation that *S. paucimobilis* induces EET through the NF‐κB → MMP signaling axis.

## 4 Discussion

In this study, we provided new insights into the pathogenesis of adenomyosis through comprehensive single‐cell transcriptomics and functional validation experiments. Our results suggested that epithelial–endothelial transition (EET) was a key mechanism in the development of adenomyosis and that *Sphingomonas paucimobilis* (*S. paucimobilis*) was a key microbial inducer of this pathological process.

Unlike the traditional epithelial–mesenchymal transition (EMT) [1] that has been widely studied in adenomyosis, EET involved the direct transformation of epithelial cells into endothelial‐like cells, which was characterized by the co‐expression of epithelial cell markers (EPCAM) and endothelial cell markers (PECAM1) [28]. In cancer biology, EET is considered a unique cell plasticity mechanism that contributes to tumor angiogenesis and metastasis [29, 30]. Recent studies have highlighted the importance of cell plasticity in various gynecological diseases [9]. Our results suggested that EET might be a common mechanism of endometrial invasion in adenomyosis and endometriosis, which might explain the frequent comorbidity of these diseases [4].

Our trajectory analysis showed that EETs undergo distinct molecular phases, with early activation of the collagen biosynthetic pathway followed by enhanced NABA matrix‐related processes [31]. This sequential activation pattern differs from EMT, in which cells typically lose epithelial features while acquiring mesenchymal characteristics [32]. The maintenance of epithelial markers during EET might explain the unique invasive capacity of adenomyosis lesions, which retain glandular architecture while displaying enhanced angiogenic potential [33]. Furthermore, the spatial distribution of EET cells, which was primarily within adenomyosis lesions, suggested that this process was driven by local microenvironmental factors rather than systemic hormonal influences [34].

We identified *S. paucimobilis* as a key driver of EET, demonstrating for the first time a bacterial‐mediated epithelial–endothelial transition in gynecological disease. *S. paucimobilis* is a Gram‐negative bacterium belonging to the family *Sphingomonas* and an increasingly recognized opportunistic pathogen [35]. The spatial localization of *S. paucimobilis* at the margins of adenomyosis lesions and its colocalization with EET cells suggest a direct causal relationship between bacterial presence and cellular transformation. Previous studies had shown that bacterial pathogens can trigger cellular plasticity programs through direct contact‐dependent mechanisms [36, 37]. Our in vitro experiments demonstrated that S. paucimobilis induced EET within 12 h of coculture, indicating a rapid interaction between bacteria and host cells.

The molecular mechanism of *S. paucimobilis*‐induced EET involved activation of the TNFα/NF‐κB signaling pathway, a well‐established inflammatory cascade that regulates cellular transformation [38, 39]. Our systematic inhibitor experiments in both in vitro and in vivo systems provided direct functional validation of this pathway. TNFα neutralization (Infliximab) and NF‐κB inhibition (Bay 11–7082) both reduced p‐p65 activation and downstream EET markers, confirming that TNFα acts upstream of NF‐κB. In contrast, MMP inhibition (GM6001) preserved NF‐κB activation but blocked downstream phenotypic changes, positioning MMPs as effectors of this signaling cascade. Importantly, our supernatant control experiment demonstrated that bacterial culture supernatant‐containing secreted metabolites but no viable bacteria—failed to induce any EET‐related changes, indicating that viable *S. paucimobilis* and likely direct host‐pathogen contact are required for pathogenesis. Activation of this pathway leads to upregulation of MMP2, which promotes extracellular matrix remodeling necessary for cell invasion [40]. Furthermore, enhanced VEGFA‐VEGFR2 signaling promotes angiogenic reprogramming, supporting the acquisition of endothelial‐like features [41]. These findings were consistent with studies of other bacterial infections in which pathogen‐induced inflammation drives epithelial plasticity and tissue remodeling [42, 43].

Although both EETs and EMT involve cellular plasticity, our data reveal distinct molecular signatures and functional outcomes. EMT typically involves loss of E‐cadherin expression and acquisition of mesenchymal markers such as vimentin and N‐cadherin [44, 45]. In contrast, EETs retain epithelial identity while acquiring endothelial features, resulting in hybrid cells with distinct functional properties [45]. This distinction has important implications for understanding the pathology of adenomyosis, as EET cells retain secretory functions while acquiring invasive and angiogenic capabilities [45].

Endothelial features acquired by EETs, particularly PECAM1 expression and VEGFA production, are directly responsible for the enhanced vascularization observed in adenomyosis lesions [46]. This neovascularization supports lesion growth and may promote further bacterial colonization, thereby creating a positive feedback loop that perpetuates disease progression [47]. Furthermore, the dual epithelial–endothelial phenotype may explain the resistance of adenomyosis lesions to conventional treatments that target only epithelial or endothelial cells [48].

Recent studies have highlighted the importance of cellular plasticity in various gynecological diseases [9]. Our results suggested that EETs were a common mechanism for endometrial invasion in adenomyosis and endometriosis, which explained the frequent comorbidity of these diseases [4]. The discovery of EETs as a unique cellular transformation process opens new avenues for therapeutic intervention, particularly by targeting unique molecular pathways that regulate this transformation [49].

Our study incorporates several methodological innovations that advance the field of adenomyosis research. Single‐cell RNA sequencing combined with CSI‐Microbes analysis can identify host‐microbe interactions that were missed by traditional analytical methods [22]. Validation using 2bRAD‐M sequencing confirmed the authenticity of the microbial signals detected in the single‐cell data and addressed concerns about potential contamination artifacts [50]. However, some limitations should be acknowledged. The relatively small sample size might limit the generalizability of our findings to different patient populations. In addition, the cross‐sectional study design is not conducive to assessing the temporal relationship between bacterial colonization and disease progression. Future longitudinal studies are needed to determine the sequence of events during the development of adenomyosis.

In summary, our study establishes EET as a novel pathogenic mechanism of adenomyosis and identifies *S. paucimobilis* as a key microbial inducer of this process. These findings fundamentally deepen our understanding of the pathogenesis of adenomyosis and provide new directions for diagnostic and therapeutic approaches. The demonstration that specific bacterial infections can drive cellular transformation processes has broader implications for understanding the role of the microbiome in gynecological disease and opens new avenues for microbiome‐based interventions in women's health.

## 5 Conclusion

This study identifies epithelial–endothelial transition (EET) as a novel pathogenic mechanism in adenomyosis and reveals this process is driven by *Sphingomonas paucimobilis*. Through integrative multi‐omics analysis, we demonstrate that *S. paucimobilis* induces EET by activating the TNFα/NF‐κB signaling pathway, which in turn upregulates MMP2 and the VEGFA‐VEGFR2 pathway, promoting the transformation of epithelial cells toward an endothelial‐like phenotype. These findings establish a direct link between a specific microorganism and cellular transformation in adenomyosis, providing a new paradigm for disease diagnosis and treatment. The identification of *S. paucimobilis* as a causative agent suggests the potential for antimicrobial therapies, while the discovery of the EET pathway offers new biomarkers and targets for precision medicine. This work fundamentally advances our understanding of host‐microbe interactions in gynecological diseases.

## Author Contributions

X.Y and G.L. orchestrated the study and contributed to its design. P.C. was responsible for the data analysis, manuscript drafting. H.S. managed the animal husbandry, conducted the dissections and sampling. J.H., P.C., and Y.Z. managed the cell experiments. X.L. facilitated the human and animal ethics application process and supported the sampling efforts. X.L., X.Y., and G.L. provided financial support, oversaw the research project, and contributed to manuscript revisions. All authors have read and endorsed the final version of the manuscript.

## Funding

This work was supported by the Natural Science Foundation of China (No. 82571898), Natural Science Foundation of Guangdong Province (No. 2023A1515012940), Key laboratory start‐up project (Sixth Affiliated Hospital of Sun Yat‐sen University) (No. 2023WST04) for their partial research support, Fertility Research Program of Young and Middle‐aged Physicians in 2024 (No. BJHPA‐2024‐SHZHYXZHQNYJ‐004) of Beijing Health Promotion Association donated by Merck Serono Co., Ltd. and Postdoctoral Fellowship Program of CPSF (GZC20233216).

## Ethics Statement

The experiment was approved by the Ethics Committee of the Institutional Review Board at the Sixth Affiliated Hospital of Sun Yat‐sen University and conducted in accordance with the Declaration of Helsinki (2020ZSLYEC‐281), and the Animal Ethics Committee of the Sixth Affiliated Hospital of Sun Yat‐sen University in accordance with the Guide for the Care and Use of Laboratory Animals (IACUC‐2026011601).

## Conflicts of Interest

The authors declare no conflicts of interest.

## Supporting information




**Supporting File**: advs74812‐sup‐0001‐SuppMat.docx.

## Data Availability

The raw sequence data reported in this paper have been deposited in NODE (https://www.biosino.org/node) by pasting the accession (OEP004165) into the text search box.
