# Supplementary material for: Sphingomonas paucimobilis‐Driven Epithelial–Endothelial Transition in Adenomyosis Pathogenesis
Source: Adv Sci (Weinh). 2026 Mar 20;13(29):e16652. doi: 10.1002/advs.202516652 (PMC13205861; doi:10.1002/advs.202516652)
Supplement: Supplementary file 1 — Supporting File: advs74812‐sup‐0001‐SuppMat.docx. [file ADVS-13-e16652-s001.docx]

***Sphingomonas paucimobilis*-Driven Epithelial-Endothelial Transition in Adenomyosis Pathogenesis**

Peigen Chen^1,2,3^, Hao Shi^1,2,3^, Junxian He^1,2,3^, Ziyu Liu^1,2,3^, Yuanyuan Zhu^1,2,3^, Xiaoyan Liang^1,2,3^, Guihua Liu^1,2,3, #^, Xing Yang^1,2,3,^ ^#^

1. Reproductive Medicine Center, The Sixth Affiliated Hospital, Sun Yat-sen University
2. GuangDong Engineering Technology Research Center of Fertility Preservation
3. Biomedical Innovation Center, The Sixth Affiliated Hospital, Sun Yat-sen University

#Email: Xing Yang (yangx52@mail.sysu.edu.cn); Guihua Liu ([liuguihua@mail.sysu.edu.cn](mailto:liuguihua@mail.sysu.edu.cn))


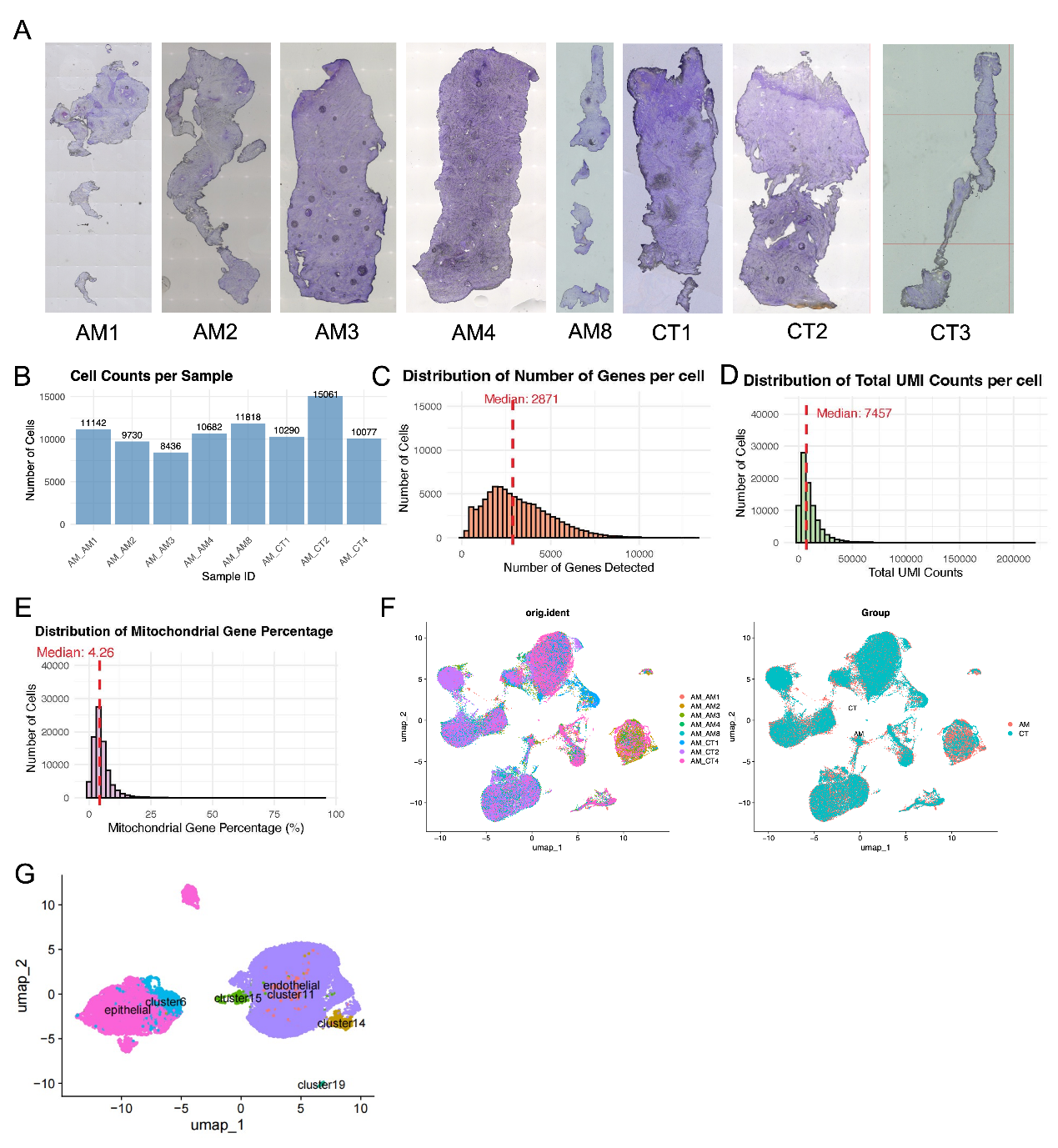


**Supplementary Figure S1. Quality control and integrated analysis of single-cell RNA sequencing data**

1. HE-stained images of samples included in the testing;

B-E. Quality control metrics for single-cell RNA sequencing data, including the number of genes per cell, UMI counts, distribution of mitochondrial gene percentages, and statistics of 87,236 high-quality cells retained after rigorous quality control;

E. The Harmony algorithm integrates UMAP plots before and after data from 8 samples, showing effective removal of batch effects;

F. Detailed procedure and quality assessment of epithelial and endothelial cell recolonization analysis.


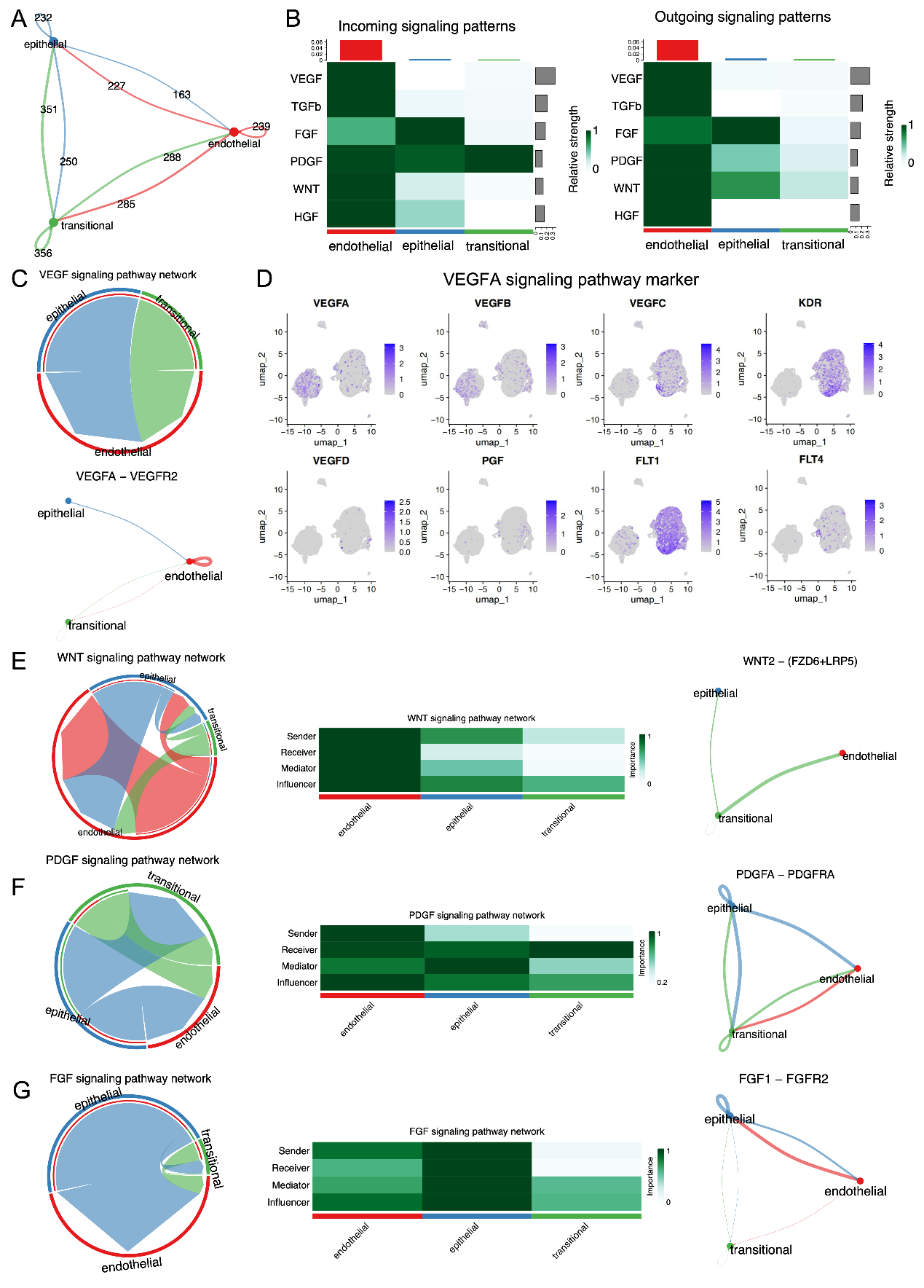


**Supplementary Figure S2. Intercellular communication network analysis**

1. Global communication network diagram between epithelial, endothelial and transformed cell populations, showing transformed cells as key intermediaries connecting epithelial and endothelial compartments;
2. Input and output signaling pattern analysis showing different communication characteristics: epithelial cells mainly act as signal senders, endothelial cells both send and receive signals, and transformed cells exhibit the highest signal-receiving capacity;
3. VEGF signaling pathway analysis shows the highest activity among all analyzed pathways, sending signals mainly from transformed cells to endothelial cells;
4. UMAP visualization of VEGF pathway components;
5. Complex bidirectional communication pattern of the WNT signaling network with specific expression of WNT2 in transformed cells;
6. PDGF and FGF signaling pathways show strong activity mainly in epithelial and transformed cell populations.


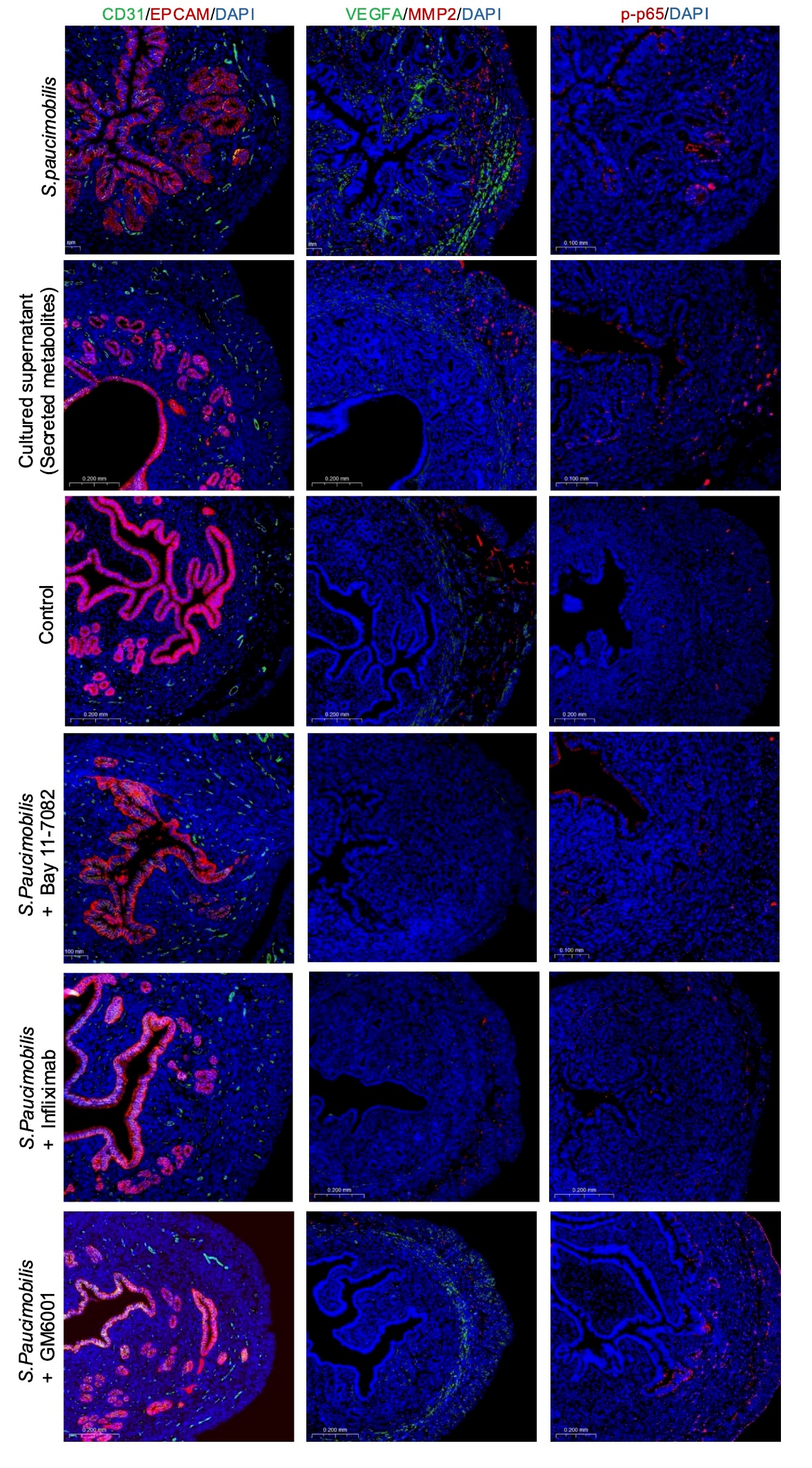


**Supplementary Figure S3. Immunofluorescence validation of EET markers in the 6-group mouse experiment**

Representative immunofluorescence images for EPCAM, CD31/PECAM1, MMP2, VEGFA, and p-p65 in uterine tissue sections from all 6 experimental groups (n=5 mice per group). Scale bars: 100 μm (low magnification), 20 μm (high magnification). Quantitative validation of these markers at the transcriptomic level is provided in Fig. 4D-G.


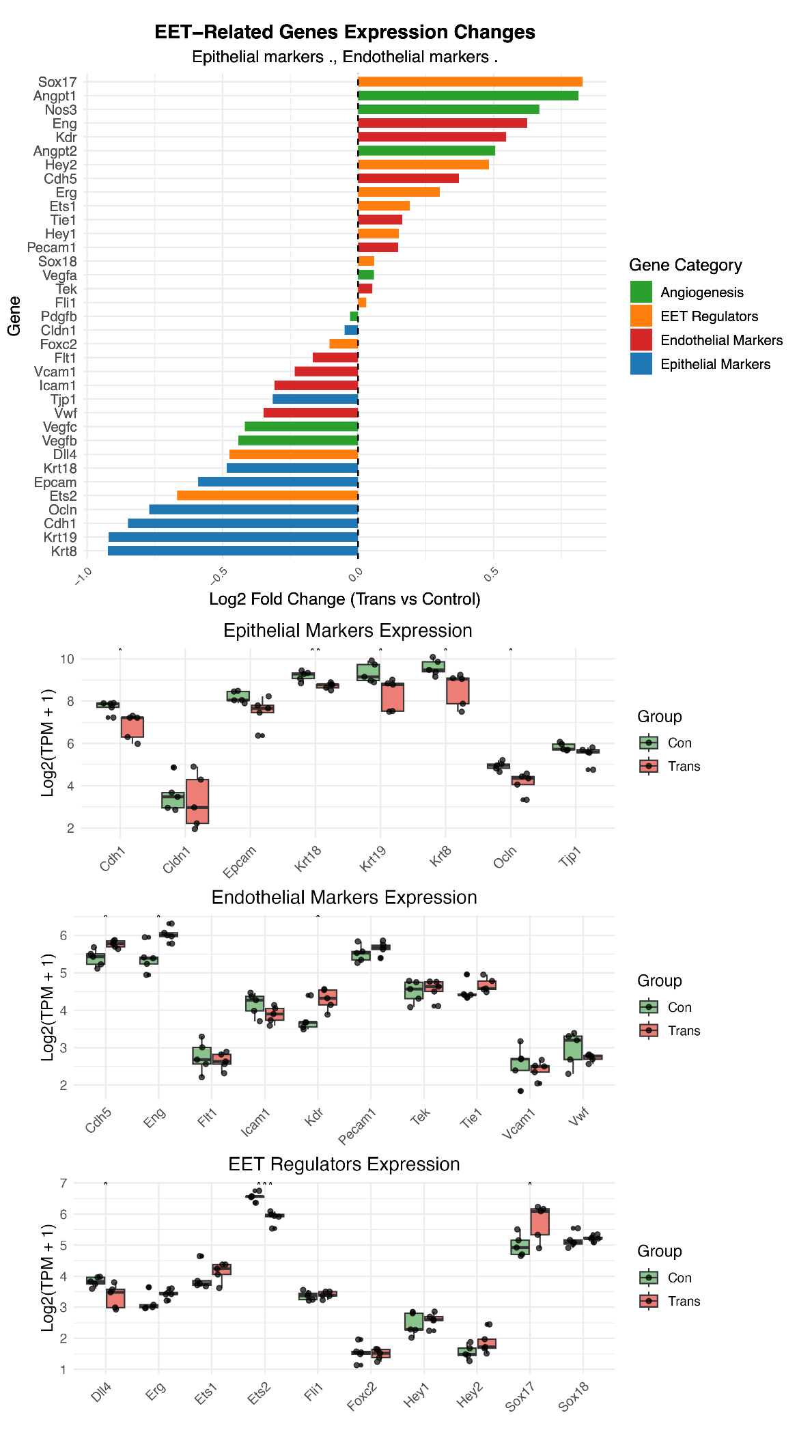


**Supplementary Figure S4. Analysis of EET-related gene expression in mouse models**

Detailed analysis of mouse model transcriptome data (n=5 mice per group) reveals a molecular signature of EET consistent with human findings, including patterns of up-regulation of endothelial-related genes and down-regulation of epithelial markers. Data are presented as mean ± SD. Statistical analysis: DESeq2 for differential expression analysis. *p<0.05, **p<0.01, ***p<0.001.

**
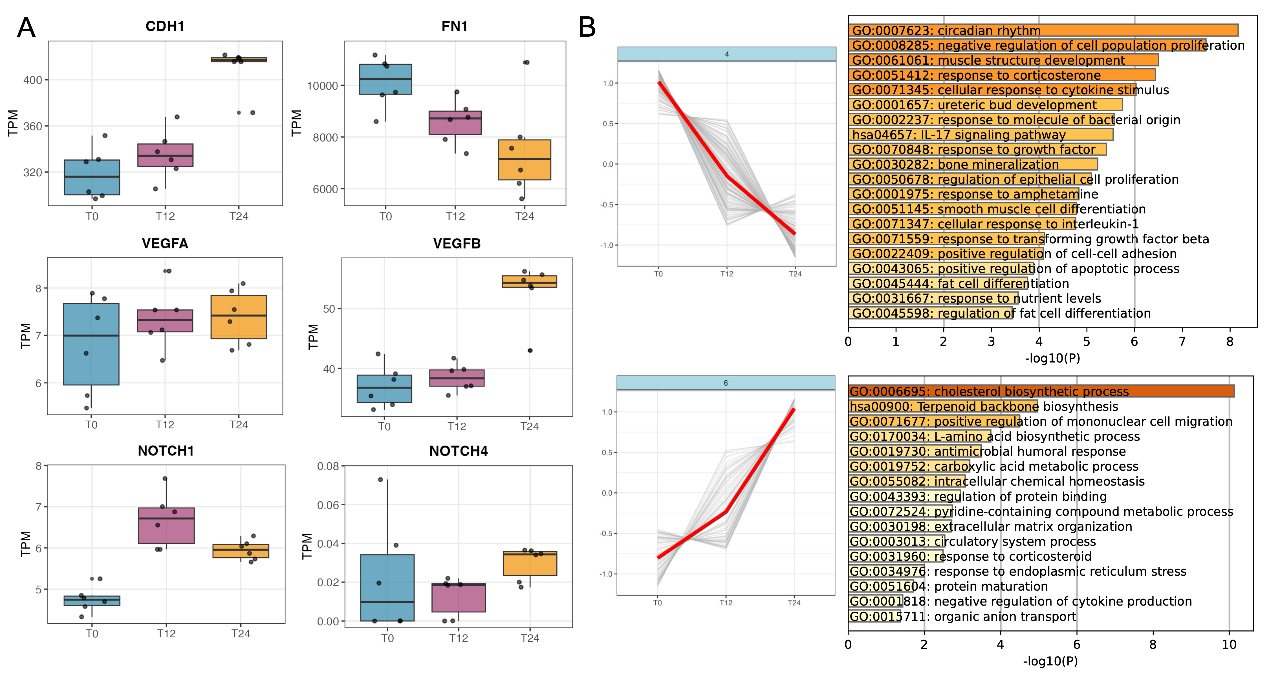
**

**Supplementary Figure S5. Comprehensive transcriptomic marker analysis in in vitro experiments**

A. Integrated marker analysis of cell transformation time course. Epithelial markers (EPCAM, CDH1, KRT8) were progressively downregulated, while endothelial markers (PECAM1, VWF, CD31) and EET-related genes (MMP2, VEGFA, SNAI1) were upregulated simultaneously;

B. Mfuzz clustering analysis grouped genes according to temporal expression patterns, revealing early response genes (rapid induction within 12 hours) and late response genes (peak expression at 24 hours). Functional enrichment analysis revealed that early responses involved diminished cell-cell adhesion and disassembly of epithelial junctions, while late responses were characterized by enhanced cell proliferation, monocyte migration, and cholesterol biosynthetic processes.


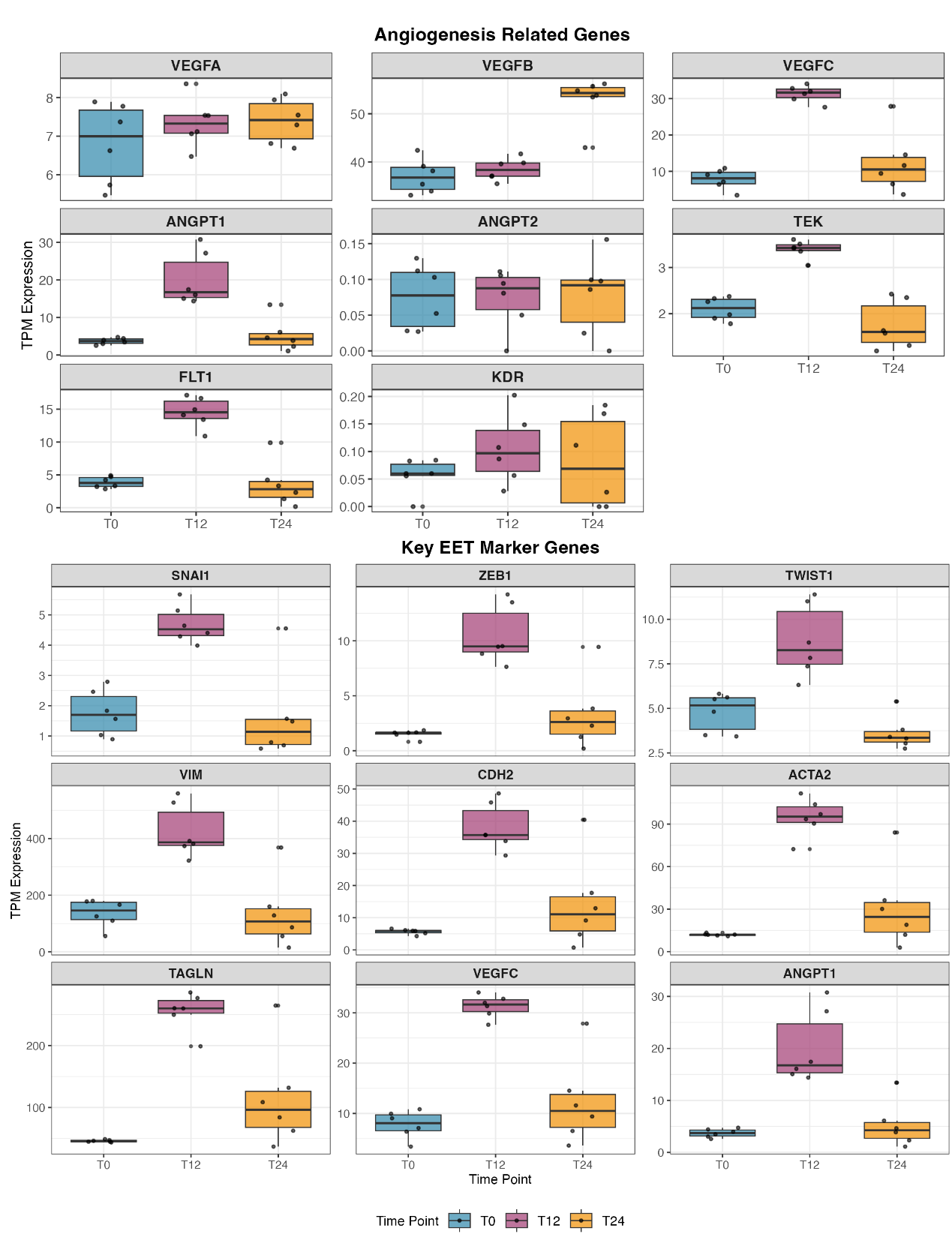


**Supplementary Figure S6. Time-series gene expression analysis in in vitro experiments**

Detailed expression kinetic analysis of cell transformation markers at different time points: Time-dependent expression changes of EET-related transcription factors and regulators, cell cycle and proliferation-related genes.


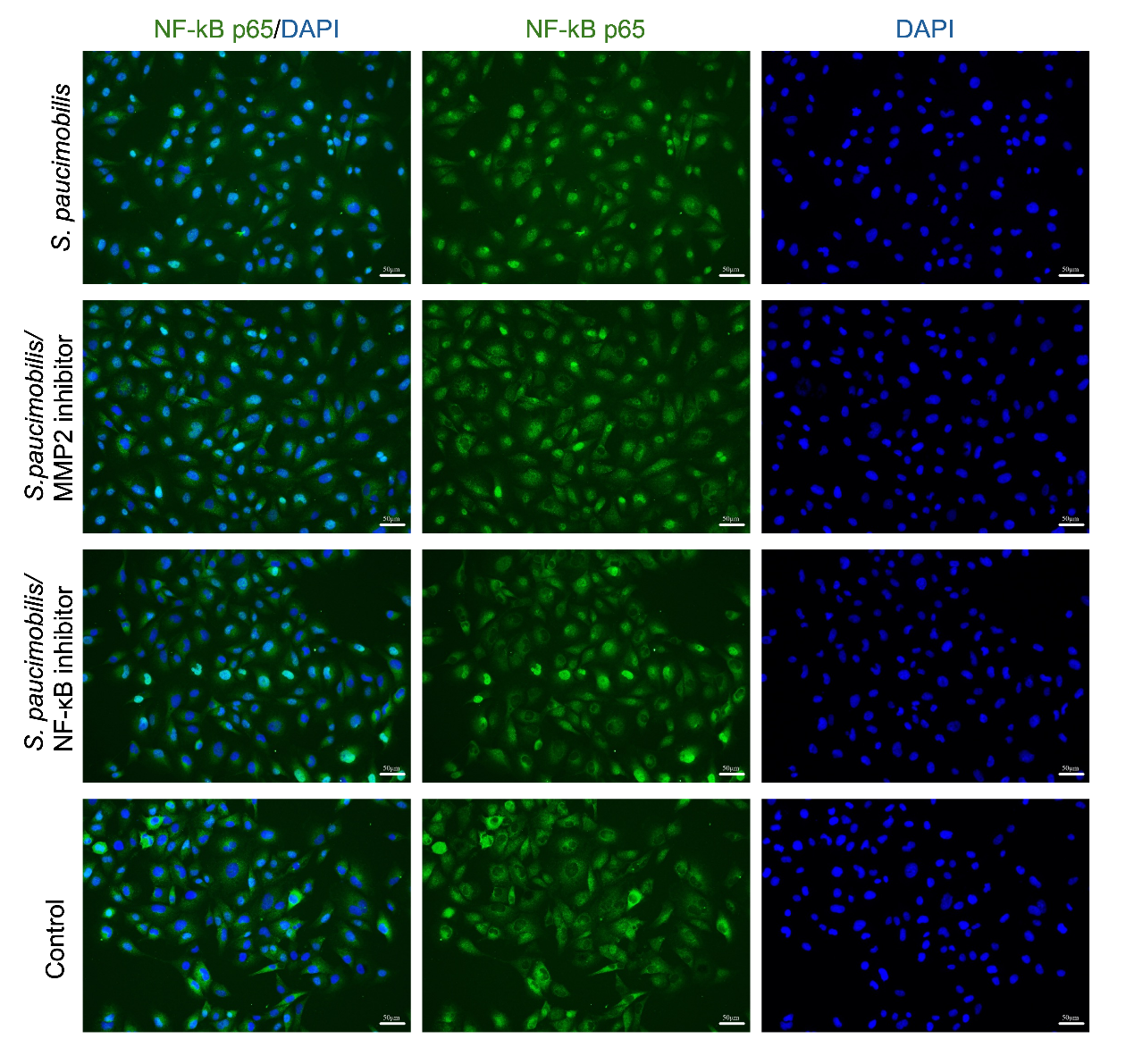


**Supplementary Figure S7. Immunofluorescence analysis of p65 nuclear translocation**

Representative immunofluorescence images showing p65 (red) localization at 6 hours post-co-culture across the 4 experimental groups (Ctrl, Live, Live + NF-κB inh, Live + MMP inh). DAPI (blue) indicates nuclear staining. The Ctrl group shows predominantly cytoplasmic p65 localization, while the Live group exhibits robust nuclear accumulation. NF-κB inhibitor treatment blocks p65 nuclear translocation, while MMP inhibitor treatment does not affect p65 localization. Scale bar: 20 μm. Quantification of p65 nuclear-to-cytoplasmic ratio is shown in Fig. 5F (n=3 independent experiments, ≥50 cells quantified per group per experiment; one-way ANOVA with Tukey's post-hoc test, ***p<0.001).


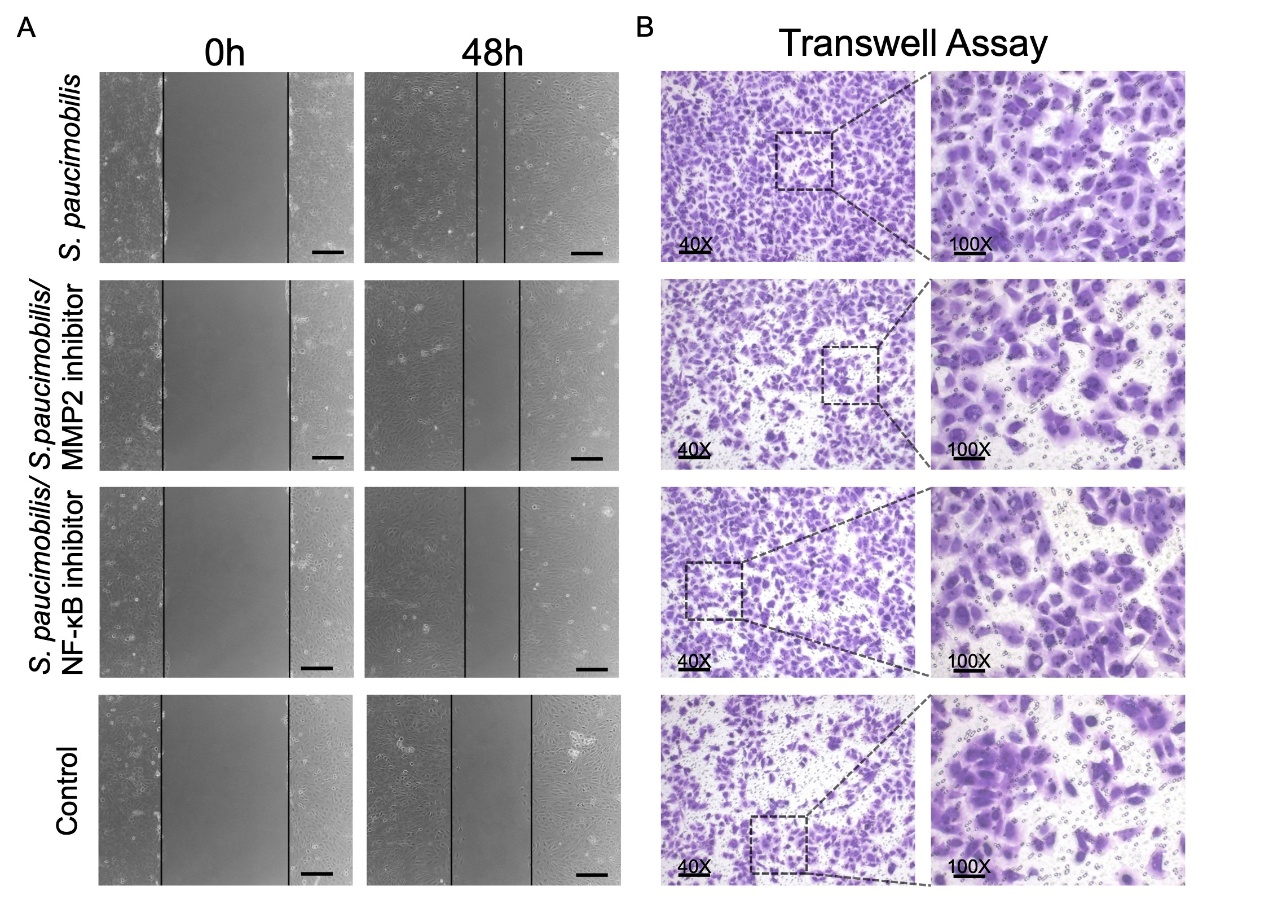


**Supplementary Figure S8. Functional validation of *S. paucimobilis*-induced cell migration**

A. Representative images from wound healing scratch assay at 0h and 24h across the 4 groups. Black dashed lines indicate wound edges. White dashed boxes indicate regions of interest. Scale bar: 200 μm. Quantification is shown in Fig. 5G.

B. Representative images from Transwell migration assay across the 4 groups. Left panels: low-magnification overview (scale bar: 200 μm) with white dashed boxes indicating regions of interest. Right panels: 4× enlarged view of boxed areas (scale bar: 50 μm). Crystal violet staining of migrated cells. Quantification is shown in Fig. 5H.

All experiments were performed in n=3 independent experiments. Data are presented as mean ± SD. Statistical analysis: one-way ANOVA with Tukey's post-hoc test. *p<0.05, **p<0.01, ***p<0.001.
